# Carbon Nanotube Hydrogels Reveal Threshold‐Dependent Regulation of Neuroblastoma Cell Growth and Maturation by Mechanical and Chemical Factors

**DOI:** 10.1002/smsc.202500401

**Published:** 2025-09-18

**Authors:** Bahaa Daou, Maurizio Prato, Sonia Alonso‐Martín, Nuria Alegret

**Affiliations:** ^1^ Center for Cooperative Research in Biomaterials (CIC BiomaGUNE) Basque Research and Technology Alliance (BRTA) 20014 Donostia/San Sebastián Spain; ^2^ Stem Cells and Aging Group Bioengineering Area Biogipuzkoa Health Research Institute 20014 Donostia/San Sebastián Spain; ^3^ Ikerbasque, Basque Foundation for Science 48013 Bilbao Spain; ^4^ Department of Chemical and Pharmaceutical Sciences Universitá Degli Studi di Trieste 34127 Trieste Italy; ^5^ CIBERNED, ISCIII (CIBER de Enfermedades Neurodegenerativas, Instituto de Salud Carlos III) 28031 Madrid Spain; ^6^ Cardiac Diseases Group Systemic Diseases Area Biogipuzkoa Health Research Institute 20014 Donostia/San Sebastián Spain

**Keywords:** carbon nanotubes, dynamic crosslinkings, hydrogels, nerve regenerations, neural interfacing, phase inversions, tissue engineering

## Abstract

Carbon nanotube (CNT)‐based hydrogels have the potential to serve as 3D platforms for nerve regeneration. However, the interplay between different cues governing the formation of a complex tissue‐like cellular structure is still ambiguous. Herein, two approaches are adopted to develop PVA/CNT hydrogels using phase inversion method and low kinetic gelation, enabling unprecedented CNT loading capacity (75% w/w) without compromising their elasticity. By controlling key factors affecting cell coverage and maturation, including Young's modulus (YM), CNT concentration, and pore size, distinct thresholds are identified where these factors dominate cell coverage. Results demonstrated that when CNT exceeds 60% w/w or a coating is applied to enhance CNT–cell interaction, CNT effect dominates, increasing cell coverage with increasing CNT concentration. However, below a specific YM threshold, YM dominates cell growth, covering up to 50% of the scaffold surface regardless of CNT concentration or exposure. Lastly, controlling the pore size to 100–250 μm further increased cell coverage to >70%, breaking through previous plateau and upregulating TUBB3 maturity marker. Additionally, certain key factors are seen to synergistically codominate in determining cell growth.

## Graphical abstract

Poly(vinyl alcohol)/carbon nanotube (PVA/CNT) hydrogels with unprecedented CNT loading (75% w/w) were engineered as 3D scaffolds for nerve regeneration. By tuning Young's modulus (YM), CNT concentration, and pore size, distinct thresholds controlling cell coverage were identified. Optimized scaffolds achieved > 70% coverage and upregulated β‐3‐tubulin (TUBB3), highlighting synergistic cues driving neuronal maturation.

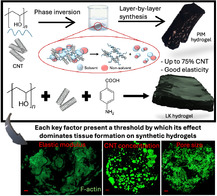

## 1 Introduction

Upon peripheral nerve injury, various biological mechanisms take place to return homeostasis to the nerve tissue.^[^ ^1^ ^]^ In summary, after 1 h of injury, calpains cleave neurofilaments, and die back starts. Conduction through the severed nerve is still mediated after 24 h, however, degeneration occurs in the proximal stump till the first healthy node of Ranvier.^[^ ^1^ ^]^ Daughter axons are pruned, but axonal sprouts are favored, this gives rise to bifurcations in the regenerated nerve tissue.^[^ ^1^ ^]^ In the distal stump, Wallerian degeneration happens within the 1st week as a result of signal absence from the spinal cord. It is worth mentioning that Schwann cells during the 1st week after injury clear up cell remnants via phagocytosis, which allows healthy regeneration.^[^ ^1^ ^]^ Finally, stem cell recruitment is observed alongside increased neurofilament expression and Schwan cells often undergo de‐differentiation to allow cell division in order to myelinate new axonal sprouts.^[^ ^2^, ^3^, ^4^ ^]^

In some instances, the regenerative process can be overwhelming to the tissue, thus a glial cavity is formed due to the activation of the regenerative process. This cavity is predominantly characterized by astrogliosis, migration of fibroblasts to lesion site and infiltration of inflammatory cells, leading to further axonal shortening and severe demyelination.^[^ ^5^, ^6^, ^7^ ^]^ Hence, the recovery can be limited partially or hindered completely.^[^ ^8^ ^]^

Efforts have been focused on facilitating nerve repair to avoid the formation of glial scars or neuromas, but without hindering the natural regenerative process.^[^ ^9^, ^10^ ^]^ To this end, nerve tissue engineering poses as an alternative to current treatments such as autografting, medications, and other biological approaches.^[^ ^11^ ^]^

The complexity of both central (CNS) and peripheral (PNS) nervous systems in general and the diversity of neurons specifically complicate the process of designing functional biomaterials or synthetic scaffolds that would support cell growth, promote neuronal plasticity, and the ability to form cell‐cell and cell‐matrix connections, ultimately leading to full tissue formation on top of these synthetic 3D networks.^[^ ^11^ ^]^ Various extrinsic signals affect neuronal diversity, and there can be around 10 000 types of neurons. Each one presenting diverse set of ion channels, thus equipping cells with a range of excitation thresholds and unique firing patterns.^[^ ^12^ ^]^ This complexity is translated to the Eetracellular matrix (ECM), which frequently remodels itself through various mechanisms, including endocytosis and resurfacing of some matrix glycoproteins, such as Tenascin‐R, found in perineuronal nets and perisynaptic ECM. Notably, similar processes are seen with other glycoproteins such as hyaluronic acid (HA).^[^ ^13^ ^]^ Hence, engineered 3D scaffolds for nerve regeneration must accommodate for this frequent remodeling without compromising the integrity of the tissue.

Hydrogels, a tridimensional network of polymer chains engulfing water, present tunable properties, high hydrophilicity, and biomimicking properties.^[^ ^14^, ^15^, ^16^, ^17^ ^]^ Their functions can be further enhanced using additives such as carbon nanotubes (CNT).^[^ ^18^, ^19^, ^20^ ^]^

CNTs have garnered significant attention since their discovery in 1991 by Sumio Iijima,^[^ ^21^ ^]^ primarily due to their exceptional electrical and mechanical properties.^[^ ^21^, ^22^, ^23^ ^]^ Subsequently, CNT‐based 3D scaffolds have become increasingly prevalent in research, although concerns regarding cytotoxicity and biocompatibility, among other factors, have impeded their progress into clinical trials.^[^ ^22^, ^24^ ^]^ Nevertheless, with the surge of scientific research on CNT‐based materials, these concerns have been mitigated, and CNTs have demonstrated their superiority as conductive additives, promoting neuronal maturation, proliferation, and synaptic signaling.^[^ ^25^, ^26^, ^27^ ^]^ Additionally, CNTs have outperformed other conductive additives in various aspects.^[^ ^28^, ^29^ ^]^

On one hand, polyvinyl alcohol (PVA)‐CNT‐based hydrogels have been reported before,^[^ ^30^, ^31^, ^32^ ^]^ However, loading high concentrations of CNTs compromises hydrogel's integrity, resulting in a brittle scaffold or degradation over time. To resolve this issue, focus must be switched more to covalent and noncovalent dynamic crosslinking, which has been proven to accommodate for stress even in the presence of additives.^[^ ^33^, ^34^ ^]^

On the other hand, 3D templating can be done to give extreme stability to a system at the expense of a higher Young's modulus (YM). One way to do that is through phase inversion or phase separation method (PIM). PIM, best described by Flory‐Huggins thermodynamic solution theory,^[^ ^35^, ^36^ ^]^ is the process by which a thermodynamically stable solution of polymer is mixed with a nonsolvent, resulting in two phases: polymer rich and polymer‐lean phases. Eventually the polymer rich phase undergoes solidification through processes like gelation, crystallization, and so on.^[^ ^37^, ^38^ ^]^

In this work, we present the synthesis of two types of PVA‐based novel hydrogels that incorporate large amount of CNTs (up to 75%). The first hydrogel (denoted by LK), with outstanding stability and CNT loading of about 50% (w/w), was obtained by noncovalent dynamic crosslinking using 4‐aminobenzoic acid. The second is based on PIM, leading to more than 75% (w/w) of CNTs loading without compromising the elasticity of the material. We have also evaluated their biocompatibility and their potential as scaffolds for neural tissue engineering with neuroblastoma SH‐SY5Y cells. Indeed, we demonstrate that Young's modulus (YM), CNTs concentration, porosity, and pore size have an impact on cell attachment, infiltration, and maturation. Together, the presented effects of CNT hydrogels lay down foundation for promoting the use of CNTs for nerve regeneration in clinical practices.

## 2 Experimental Section

### 2.1 Materials and Reagents

Multiwalled CNT (MWCNT, >95%) were purchased from Nanoamor Inc. (stock# 1237YJS: inner diameter, 5−10 nm; outside diameter, 20−30 nm; length, 0.5−2 μm), Polyvinyl Alcohol (Mw 146 000–186 000, 99+% hydrolyzed) CAS 9002‐89‐5 and 4‐Amino benzoic acid (PABA, 4ABA, Vitamin H1) CAS 150‐13‐0, CA. Gelatin powder from porcine skin (CAS #9000‐70‐8, solubility 50 mg Ml^−1^) were purchased from Sigma Aldrich. Phosphate‐buffered saline (PBS) buffer was purchased in tablets and prepared following manufacturer procedures (Sigma–Aldrich), corresponding to 10 mM phosphate buffer containing 137 mM NaCl and 2.7 mM KCl at pH 7.3.

The solvents were acquired from Carlo Erba Reagents SAS, Sabadell, Spain. All reagents and solvents were used as received with no further purification.

### 2.2 Synthesis of PVA/CNT Hydrogels Using PIM

20% (w/v) of PVA solution was prepared by dissolving 500 mg of PVA in 2.5 mL of MilliQ water (Millipore, USA) followed by incubation at 80 °C for at least 30 min. A specified amount of CNTs, to obtain a final concentration between 20% and 75% w/w with respect to PVA, was dispersed in 2.5 mL of water by sonication for 20 min. Then, the CNTs dispersion was mixed with the PVA solution, obtaining a final PVA concentration of 10% (w/v). The dispersion was kept at boiling point until PVA was completely solubilized (3–6 h).

On a clean glass slide, 1.5 mL of the PVA/CNT dispersion was smeared with the help of another clean glass slide. The glass slide was immersed horizontally in acetone for 10–20 min. Afterwards, the coated glass slide was removed from the acetone and air dried for 30 s. A second layer (1.5 mL of the PVA/CNT dispersion) was added onto the first layer and smeared homogeneously, followed by immersion in acetone overnight to ensure that PVA has completely underwent phase inversion. Once phase inversion was completed, the glass slide was removed from the acetone and then submerged in ultrapure water at room temperature for 2 h. Finally, the porous dry hydrogel film formed onto the glass was peeled gently and left for at least another 3 h inside the water to complete hydration. After this procedure, hydrogels with a thickness of ca. 500 μm were obtained (see **Table** **1** for nomenclature).

**Table 1:** Composition and nomenclature of different PIM and CP‐PIM hydrogels.

Material	PIMcntrl [%]	PIM20 [%]	PIM33 [%]	PIM40 [%]	PIM50 [%]	PIM60 [%]	PIM70 [%]	PIM75 [%]	CP‐PIM50 [%]	CP‐PIM75 [%]
**PVA** % (w/v)	10	10	10	10	10	10	10	10	10	10
**CNT** % (w/w)	0	20	33	40	50	60	70	75	50	75

CNT, carbon nanotubes; Cntrl, control; CP‐PIM, PIM hydrogels with controlled porosity; PIM, phase inversion method hydrogels; PVA, polyvinyl alcohol. Numbers indicate CNTs % (w/w).

### 2.3 Synthesis of PIM Hydrogels with Controlled Porosity (CP‐PIM)

Sieved gelatin, with particle sizes between 100 and 250 μm, was used as porogen to control the pore sizes of PIM hydrogels. To 1 mL of PVA/CNT dispersion prepared as mentioned above, 260 mg of sieved gelatin was mixed until a homogeneous solid was formed. The paste‐like material was fed to a syringe to be molded in a cylindrical shape and submerged in acetone. The material underwent phase inversion for 24 h. Then, the porogen was leached by submerging the scaffolds in 20 mL of water for 72 h at 37–40 °C, changing the water every 24 h. A self‐standing CP‐PIM50 (50% CNT, 10% PVA) and CP‐PIM75 (75% CNT, 10% PVA) hydrogels were obtained (Table 1).

### 2.4 Synthesis of Dynamically Crosslinked PVA/CNT Hydrogels (LK)

PVA/CNT dispersions were prepared by dissolving specified amounts of PVA (5%, 7.5%, 10% w/v) and CNTs in milliQ water until complete dissolution of PVA (at 95 °C for 2 h). 1.5% w/v of 4ABA was mixed under reflux and left for 3 h at 100 °C with occasional vortexing. The mixture was cooled down and incubated at 4 °C for an average time of 72 h for 5 mL of LK pregel dispersions. Longer incubation periods are necessary to achieve complete crosslinking when larger amounts of hydrogel were prepared, or when higher concentrations of CNTs were used, as the gelation kinetics are slower.

For in vitro studies, hydrogels were soaked overnight first in acetone and then in an alkaline solution (10 mM NaOH) at room temperature to neutralize their pH. The obtained self‐standing hydrogels were denoted by LK (**Table** **2**).

**Table 2:** Composition and nomenclature of LK and CP‐LK hydrogels.

Material	LK5 [%]	LK7.5 [%]	LK10 [%]	LK10/50 [%]	CP‐LK10 [%]	CP‐LK10/50 [%]
**PVA** % (w/v)	5	7.5	10	10	10	10
**CNT** % (w/w)	18	12	9	50	9	50
% (w/v)	1	1	1	10	1	10
**ABA** % (w/v)	1.5	1.5	1.5	1.5	1.5	1.5

ABA, amino benzoic acid; CNT, carbon nanotubes; CP‐LK, LK hydrogels with controlled porosity; LK, crosslinked PVA/CNT hydrogels; PVA, Polyvinyl Alcohol. Numbers indicate PVA % (w/v)/CNTs % (w/w).

### 2.5 Synthesis of LK Hydrogels with Controlled Porosity (CP‐LK)

Following the same procedure to synthetize LK hydrogels explained above, gelatin was introduced to the PVA/CNT/4ABA mixture. However, due to the softer nature of these hydrogels and to ensure effective crosslinking, 50 mg of sieved gelatin per 1 mL of (PVA + MWCNT + 4ABA) dispersion was mixed in a disposable 15 mL plastic container. The paste‐like material was fed to a syringe to be molded in a cylindrical shape and left at 4 °C to gelate completely for 1 week, followed by leaching of porogen in 20 mL of water at 37–40 °C for 72 h, changing the water every 24 h.

Similarly, for in vitro studies, and following the same procedure above, hydrogels were thereafter soaked overnight first in acetone and then in an alkaline solution (10 mM NaOH) at room temperature to neutralize the pH. The obtained self‐standing hydrogels were denoted by CP‐LK (Table 2).

### 2.6 4‐Point Probe (4‐PP) Measurements

Triplicates of 6 × 1 mm of each hydrogel were presoaked in PBS or water for at least 72 h to reach equilibrium, followed by measuring sheet resistance using Ossila 4‐PP machine. The target current was set up in a range between 10 and 100 μA using 10 V as maximum voltage. Conductivity was automatically calculated by specifying the thickness of each hydrogel using a digital micrometer.

### 2.7 Thermal Gravimetric Analysis (TGA)

TGA was performed under nitrogen atmosphere (25 mL·min^−1^ flow rate) using a TGA discovery (TA Instruments). Samples were equilibrated at 100 °C for 20 min and then heated at a rate of 10 °C·min^−1^, in the range from 100 to 800 °C. Measurements of at least two repetitions were recorded, and Trios V4.4.0.41128 (TA instruments) software was used to analyze the data. The corresponding experimental CNTs content was estimated from the TGA plots taking, 500 °C as the reference, according to the following equation

(1) $$\text{Experimental CNT \%}=\frac{\text{Weight at }500\;{}^{\circ}C-13\text{\% of Initial Weight }\left(\text{PVA Residue}\right)}{\text{Initial Weight}}\times 100$$

### 2.8 Fourier Transform Infrared Spectroscopy (FTIR)

FTIR was performed on a ThermoScientific FTIR spectrometer in attenuated total reflectance mode at room temperature. FTIR spectra were recorded by accumulation of 32 scans and a resolution of 4 cm^−1^.

### 2.9 Swelling

Hydrogel discs of 8 mm in diameter were immersed in MilliQ water after synthesis for at least 48 h (for PIM) or 72 h (for LK) to reach equilibrium. Then, they were weighed (Ws, g), and the height and diameter measured using an accurate digital micrometer, which were used to calculate the volume when wet (*Vs*, mm^3^). To dry them, the scaffolds were first placed at –20 °C for 24 h, then at –80 °C overnight and then lyophilized using an Alpha 2–4 LSCplus part no. 102142 at a condenser temperature between –80 °C and –92 °C with a vacuum of 0.16 Pa. When dried, they were weighed (Wd, g), and their volume measured again (Vd, mm^3^). This protocol was seen to decrease the possibility of frost‐shock and collapsing the structure of the gels. The swelling behavior is then calculated according to the following equation

(2) $$Sw\%=\frac{Ws-Wd}{Wd}\;\times 100\;Sv\%=\frac{Vs-Vd}{Vd}\;\times 100$$

Later, they were immersed in PBS to mimic physiological conditions and incubated at 37 °C for 2, 4, 24, and 48 h. The swelling in PBS is calculated at each time point according to the above equation.

### 2.10 Porosity

The porosity (*p*) was calculated following above‐described protocol for swelling and then computed using the following equation

(3) $$\%p=\frac{Ws-Wd}{\rho \;H2O\times Vs}\times 100$$

wherein *Ws*, *Wd*, and *Vs* are as defined above, and *ρ* is the water density (1 g.mL^−1^).

### 2.11 Rheology Analysis

Frequency sweep was carried out for all samples premolded to disks of an 8 mm diameter in a MCR 301 Rheometer (Anton Paar), using patterned plate geometry (diameter 8 mm) at room temperature at a fixed strain of 0.016%. First, all samples were submitted to an amplitude sweep tests to establish the linear viscoelastic region, where both the storage (G’) and the loss modulus (G’’) are independent of the applied strain at a fixed frequency of 10 Hz.

### 2.12 Pore Size and Topography

To measure the pore size and topography of the hydrogels, a scanning electron microscope (SEM) JSM‐IT800HL JEOL (Tokyo, Japan) equipped with a field emission gun and stage bias option called beam deceleration (BD) with secondary electrons detectors, backscattered electrons detector, and transmitted beam electrons detector was used. The scaffolds were first placed at −20 °C for 24 h, then at −80 °C overnight, and finally, lyophilized. After lyophilization, the volume of the hydrogels decreased noticeably. Therefore, after volume swelling measurements, a correction factor was applied as follows

(4) pore size (μm)=dried scaffold pore size x (correction factor)

wherein correction factor = 1+ (% swelling (*Sv%)*/100). Images in SEM were acquired using only SE detector and an acceleration of 5 kV for all samples except for samples with high CNTs content (PIM75, and LK10/50) where an acceleration voltage of 0.5 kV was used allowing for less charging events to occur.

### 2.13 Mechanical Properties and YM

Uniaxial compression tests were performed using a universal testing machine (Instron) where a strain–stress curve is plotted. Hydrogels were biopsy‐punched into 8 mm disks and measure, and YM was calculated by measuring the slope of the curves’ first linear portion. For the measurements of nerve tissues: brain, spinal cord, and sciatic nerve of Sprague‐Dawley rats and C57BL/6J mice were harvested and directly measured at a constant speed of 10 mm min^−1^.

### 2.14 Cell Culture and Seeding

Prior to cell seeding, all the synthesized hydrogels were placed at −20 °C for 24 h, then at −80 °C overnight, and finally, lyophilized. Then, the hydrogels were coated with 0.1 mg/mL of Poly‐D‐Lysine (PDL) for 48 h at 60 °C, which can be attributed to hydrogen‐bonds forming between the matrix (PVA)/4ABA and PDL, a notably stickier hydrogel was obtained.

Neuroblastoma SH‐SY5Y cell line was purchased from ATCC‐LGC (Cat. ATCC CRL–2266, Homo sapiens, metastatic neuroblastoma from a 4‐year‐old female, thrice cloned, RRID: CVCL_0019) and cultured in Dulbecco's Modified Eagle Medium (DMEM, GIBCO) premixed with Nutrient Mix F12 Ham media (1:1 ratio), containing 2 mM L‐glutamine (Gibco), 100 U·mL‐1 penicillin, 100 μg·mL‐1 streptomycin (Gibco), 0.5 mM sodium pyruvate, and 10% heat‐inactivated fetal bovine serum (FBS, Gibco) at 37 °C and 5% CO_2_ in tissue culture‐treated 175 cm^2^‐flasks (Nunc). For passaging, cells were detached from the flasks by incubation at 37 °C with trypsin‐EDTA solution 1X (Sigma) and spun at 480 RCF for 5 min; the obtained pellet was resuspended in 1 mL of complete media. For cell counting, the cell suspension was diluted 1:2 in the exclusion dye Trypan Blue solution (Sigma) and 10 μL of the diluted cell suspension was counted with the automated cell counter (Countess, Invitrogen). Cells were generally passed between 5 to 10 passages before seeding on top of the hydrogels.

### 2.15 Lactate Dehydrogenase (LDH) Cell Cytotoxicity Assay

The cytotoxicity of cells adhered to the scaffolds was evaluated with a modified LDH CytoTOX96 nonradioactive cytotoxicity assay kit (Promega), reported by Ali‐Boucetta et al.^[^ ^39^ ^]^ 50 μL of the supernatants were transferred to replicate wells in a 96‐well flat‐bottom plate. For LDH detection, 50 μL of substrate mix was added to the supernatants and incubated in the dark for 30 min. The reaction was terminated by the addition of 50 μL of stop solution (acetic acid). Absorbance measurements at 492 nm were taken in a microplate spectrophotometer (GeniosPro, Tecan). A 50 μL of complete medium was used to read and subtract the background from FBS. All the collected data was represented as mean of ±SD of 3 independent experiments (*n* = 3) and normalized to the average value of control wells. One‐way Anova was performed to draw statistical comparisons. Differences were considered statistically significant at *p *< 0.05.

### 2.16 Immunofluorescence

After 3 or 10 days of incubation, hydrogels with cells were fixed with 4% paraformaldehyde for 2 h at 4 °C, followed by PBS washing. Cells were permeabilized and blocked with a solution containing 0.5% Triton X‐100, 5% bovine serum albumin, and 0.02% sodium azide (NaN_3_) in PBS 1X for 2 h at 4 °C. Then, samples were incubated with antibeta‐III tubulin (TUBB3) monoclonal antibody conjugated to Alexa Fluor 647 (AF647, 1 μg·mL‐1 dilution, EP1569Y, Abcam Cat. No. ab190575 Lot: GR3407186‐2) and Actin Green 488 Ready Probes Reagent (1:10 dilution, Invitrogen Cat. No. R37110) in blocking solution as diluent at 4 °C overnight in the dark. Nuclei were stained for 1 h with 50 nM 2‐(4‐amidinophenyl)‐1H‐indole‐6‐carboxamidine (DAPI) (Sigma Aldrich CAS. 28718‐90‐3) in PBS.

For confocal imaging, hydrogels with immunostained cells were covered with PBS in a 50 mm‐diameter ≠1.5 optical glass bottom dish (Cellvis). 500−1000 μm‐Z‐stack images were taken in a light scanning microscope (Zeiss LSM 900) with Airy Scan 2, employing the excitation/emission wavelengths of 633 nm/650−735 nm for TUBB3‐AF647, 488 nm/500−600 nm for Actin Green 488, and 405 nm/415−475 nm for DAPI.

For image processing, maximum intensity Z‐projection images were generated using ZEN 2.3 software. For the quantification of cells area, fluorescence images were analyzed using ImageJ program (National Institutes of Health, USA) version Java 13.0.6. In the analysis, cell area (from green filter) was selected automatically using the software, the area of the whole gel was selected manually, and the data thereof, was reported as the ratio of cell area to total area. For cellular quantification, automatic selection of actin+ and TUBB3+ cells was carried out, the value of the raw integrated intensity was recorded, and the values were reported as the ratio of TUBB3 to actin. All the collected data was represented as means of triplicates of 3 independent experiments (*n* = 3) ±SD. One way ANOVA was performed to draw statistical comparisons between groups. Differences were considered statistically significant if *p *< 0.05.

## 3 Results and Discussion

### 3.1 Synthesis and Characterization of PIM Hydrogels

**Figure 1:**
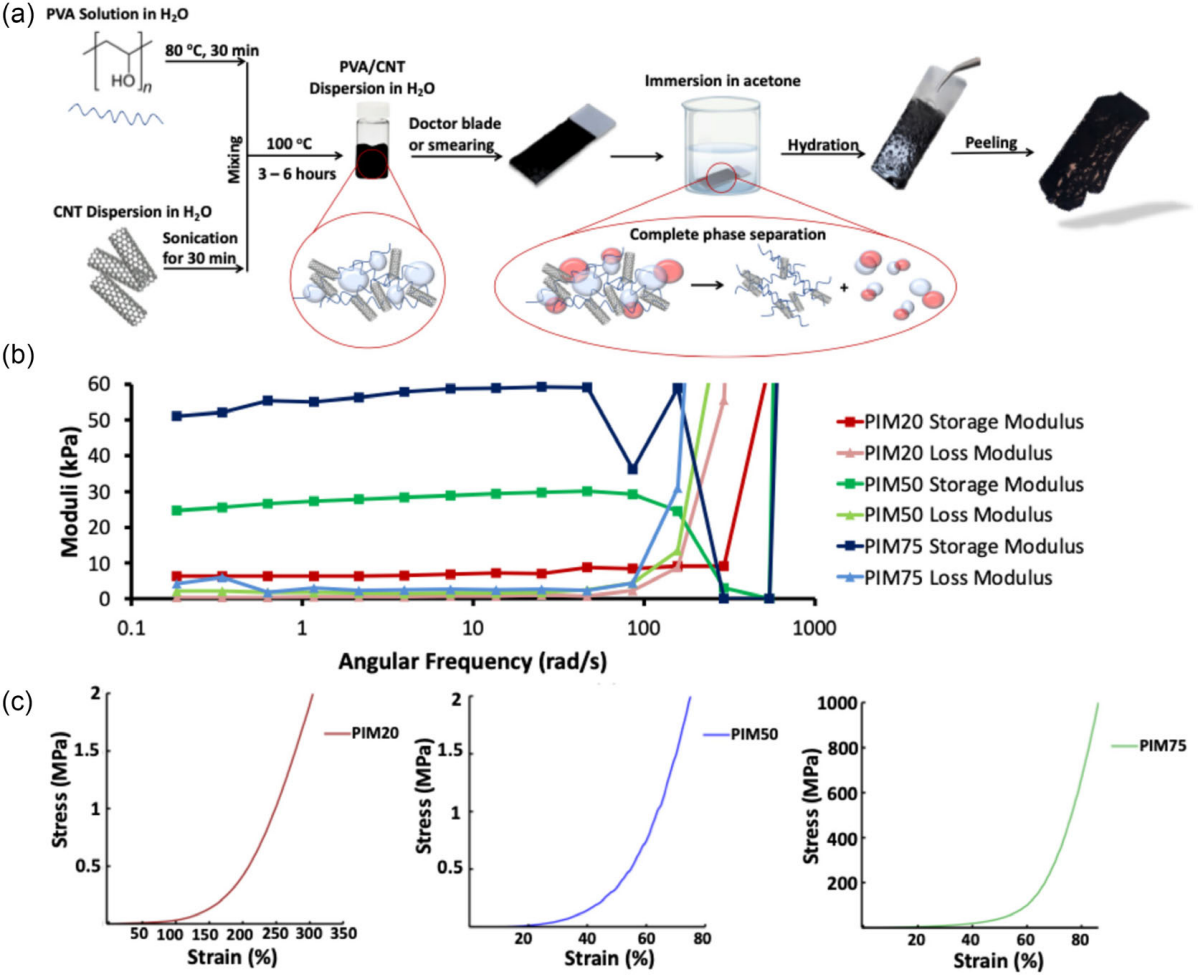
Characterization of PIM Hydrogels. a) Schematic representation of the synthesis route of PIM hydrogels. b) Frequency sweep carried out at 0.016% strain for PIM hydrogels with various CNT concentration. c) Uniaxial compression tests at constant compression ramp.

PIM hydrogels were synthesized using a three‐step method summarized in (**Figure** **1**a) and completely characterized as outlined in **(**Figure S1–S4, Supporting Information). In the presence of CNT, phase inversion occurred at a slower rate as PVA wraps around the CNTs via van der Waals forces and π–π interactions.

Pure PVA PIM hydrogel showed higher degree of shrinkage on the glass substrate upon phase inversion compared to CNT‐containing PIM hydrogels, which allowed the gel to resist shrinkage, most probably attributing this advantage to the structural stability that CNT promotes. The resistance to shrinkage upon phase inversion makes the process much more controlled and therefore allowed the PVA chains to modify themselves structurally, accommodating for up to 75% w/w of CNT without losing elasticity.

PIM hydrogels presented high storage moduli for all CNTs concentrations (Figure 1b and Figure S2a, Supporting Information). Since all hydrogels possess same concentration of PVA and since swelling is not a predominant variable, CNTs played the major role in determining the difference in the rheological properties of the PIM hydrogels. As the concentration of CNTs increase from 20% to 50%, the storage modulus increases by a factor of 4.45x from 6.6 to 29.4 kPa, respectively. Whereas, increasing CNTs concentration to 75% (PIM75) further doubled the storage modulus to 58.8 kPa (Figure 1b). The ability for the hydrogels to store up energy while maintaining a similar loss modulus G’’, can be attributed not only to CNT's effect but also to the compacted PVA matrix upon synthesis via phase inversion and the nature of interaction between CNT and PVA. Additionally, PIM scaffolds after hydration behave as a typical hydrogel showing crossover point at higher angular frequencies, specifically at around 158 rad s^−1^ measuring 8.7, 21.0, and 51.0 kPa for PIM20, PIM50, and PIM75, respectively.

Given that the materials’ final intended application is the regeneration of the central nervous system and that compressive stress is a predominant mechanism of injury in neural tissue, we have evaluated the mechanical properties of the scaffolds via compression testing. We believe this approach provides a more physiologically relevant characterization of the materials’ mechanical behavior. Therefore, following the characterization of the rheological properties, uniaxial compression test was carried out on fresh nerve tissues; namely, whole brain, spinal cord, and sciatic nerve in *mus musculus* and *rattus*. Compression tests resulted in a j‐shaped curve for all, which is a typical behavior of biological tissues allowing them to extend elastically with minimal resistance up to a certain point after which the tissue becomes stiffer and resists deformation (Figure S3, Supporting Information). PIM gels showed the same mechanical behavior and j‐shaped curve as opposed to traditional Hookean curves (Figure 1c). This means that, at fracture point less energy is released thus yielding the material tougher. Such behavior mimics the biological tissues rendering the PIM hydrogels elastic and compression‐resistant at higher strains. Additionally, the loading is completely elastic, and the hydrogel regains lost energy exerted upon compressing the system. Due to this behavior, a hysteresis loop forms when applying cyclic loading‐unloading experiment (Figure S2b,c, Supporting Information). Therefore, it's safe to deduce that phase inversion method resulted in hydrogels with biomimicking properties.

In contrast, compression‐derived YM of PIM hydrogels was calculated (Figure 1c and **Table** **3**) showing dependance on CNT concentration. For PIM20, PIM50 and PIM75, calculated YM registered between 0.254 MPa (PIM20) to 66.23 MPa (PIM75), which is well comparable to that of peripheral nerves that is, the sciatic nerve (in rodents YM_sciatic nerve_ is around 0.5 MPa). Generally, the YM of all PIM hydrogels describe a very stiff material.

**Table 3:** Summary of PIM hydrogel properties.

Material	Experimental CNT [%]a)	YM [MPa]b)	Mean Pore size [μm]c)	Porosity [%]d)	Swelling volume [%]d)	Swelling mass [%]d)
PIM20	18.2	0.25	17	65	110	450
PIM50	48.4	1.52	10	70	40	310
PIM75	69.0	66.23	23	64	19.79	270

a) Calculated amount of CNTs and PVA at 500 °C From TGA plot, considering the 13% of PVA residue.

b) Calculated from compression tests.

c) Calculated from SEM images by the equation defined above including the correction factor.

d) Calculated from swelling analysis and liquid displacement method.

### 3.2 Synthesis and Characterization of LK Hydrogels

**Figure 2:**
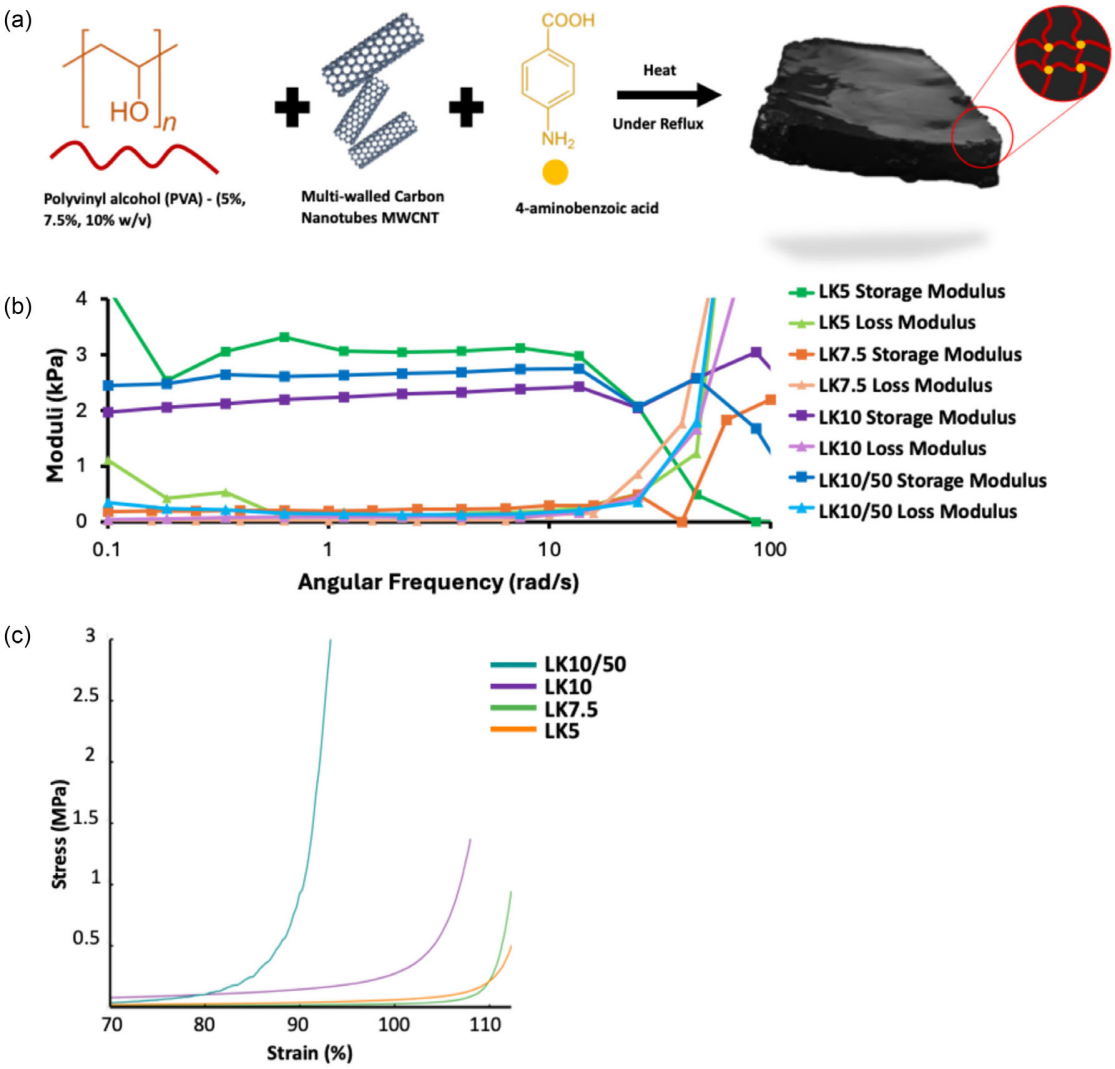
Characterization of LK Hydrogels. a) Schematic representation of the synthesis route of LK hydrogels. b) Frequency sweep carried out at 0.016% strain for various PVA concentration. c) Strain–stress curve of uniaxial compression test using universal testing machine.

Chemical crosslinking offers a pool of variables to tune the properties of the end material. We have tested various crosslinkers based on phenolic acids and other H‐rich donor/acceptor molecules, namely, Gallic Acid (GA), 4ABA, Terephthalic Acid (TPA), 5‐Aminoisophthalic Acid (5AIPA), 5,5' Dithiobis (2‐NitroBenzoic Acid) (DTNBA). We first studied an extended set of variables for each of the crosslinkers mentioned above, including crosslinker concentration, time of reaction, time of gelation, and additives (such as agar, conductive polymers, and CNTs) among others. 4ABA yielded the best mechanically and structurally stable hydrogel with the optimal conditions represented in Table 2 and according to the synthesis described in (**Figure** **2**a). Additional complete characterization was also outlined in (Figure S5 and S6, Supporting Information). The maximum limit of 4ABA concentration was 2.5% since at higher amounts it starts favoring predominantly the formation of 4ABA ‐α and ‐β crystals that inhibit the interaction with PVA.^[^ ^40^ ^]^ The final hydrogel presented lower gelation kinetics with an average of 72 h gelation time. Interestingly, with the increase of CNTs concentration, a further decrease in gelation kinetics was seen, notably with LK10/50.

Amplitude sweep on LK hydrogels was done to identify the linear viscoelastic region (LVER) (Figure S5a, Supporting Information). We identified the optimal strain value at 0.016% (higher strain yielded similar results but less stability due to dehydration) and carried out frequency sweeps using this strain value. The hydrogels registered relatively lower moduli, compared to PIM gels, reflecting their softer matrix; however, maintaining the typical hydrogel behavior with crossover points at around 1.1, 0.35, 2.75, and 2.5 kPa for LK5, LK7.5, LK10, and LK10/50, respectively (Figure 2b). For LK hydrogels, CNT played a secondary role in determining the rheological or mechanical properties of the material. PVA concentration predominantly dictated their behavior alongside swelling/water content. The storage modulus increased with higher PVA concentration true for both LK7.5 versus LK10, LK5 did not seem to follow such trend due to the synergistic effect of PVA concentration and possessing highest swelling and thus, having the highest storage modulus. In contrast, CNT effect on LK hydrogel could be seen comparing LK10 versus LK10/50 where the higher the CNT concentration, the higher the storage modulus.

Similar to PIM hydrogels, uniaxial compression tests resulted in a j‐shaped curve (Figure 2c) where PVA chains align with the stress, rendering the material stiffer at higher strains. PVA concentration is the only factor effecting YM in LK5, 7.5, and 10, where the Young's moduli increased with increasing PVA concentration, measuring 30, 70, and 200 kPa respectively. Continuing the trend of PIM gels, CNT increased the stiffness of the hydrogel. Thus, for LK10/50 the YM was seen to increase from 0.20 to 1.16 MPa (Figure 2c and **Table** **4**).

**Table 4:** Summary of various properties of LK hydrogels.

Material	Experimental CNT [%]a)	YM [MPa]b)	Pore size [μm]c)	Porosity [%]d)	Swelling volume [%]d)	Swelling mass [%]d)
LK5	12.46	0.03	12	83	38	1100
LK7.5	14.60	0.07	11	67	20	750
LK10	13.78	0.20	10	73	28	600
LK10/50	56.50	1.16	41	60	5	500

a) Calculated amount of CNT and PVA at 500 °C From TGA plot, considering the 13% of PVA residue.

b) Calculated from compression tests.

c) Calculated from SEM images.

d) Calculated from swelling analysis and liquid displacement method.

### 3.3 Pore Size Control: CP‐PIM and CP‐LK Hydrogels

All PIM and LK materials were optimized by controlling the pore size of the material *via* porogen leaching method using gelatin as porogen (Figure S7, Supporting Information). Commercially available gelatin particles have an irregular shape and size, thus the porogen was sieved to obtain homogeneous particles of sizes between 100 and 250 μm. Briefly, to obtain controlled porosity gels, we introduced gelatin in the initial PVA/CNTs mixture. The amount of gelatin particles could be varied based on the targeted pore size needed and the stability of the gel; for instance, LK hydrogels generally tolerated less amount of porogen (50 mg mL^−1^) whereas PIM hydrogels were stable even with higher amount of the porogen without jeopardizing the structure (up to 250–260 mg mL^−1^). Gelatin takes up most of the water in the PVA/CNTs dispersion when added, which makes it moldable in any shape, extrudable, and effective at any PVA or CNTs concentration (Figure S7a, Supporting Information). Upon leaching, gelatin detaches completely from the matrix, evident by visible floating semi‐translucent strands of gelatin in the water. The resulting scaffold allowed water to be released upon compression and quickly reabsorb it manifesting in a sponge‐like behavior. Morphologically, SEM images revealed that the pores adopted thicker walls due to PVA enwrapping each gelatin particle and adapting to the particle shape of gelatin (Figure S7b, Supporting Information). Additionally, the pore size distribution demonstrated that 68.1% of the pores fall within the desired values with an average of 229 ± 39.3 μm (Figure S7c, Supporting Information), whereas bigger pore‐size could be attributed to a small degree of swelling of gelatin particles upon contact with water.

### 3.4 In vitro Analysis of PIM and LK Gels Using Neuroblastoma SH‐SY5Y Cells

All in vitro experiments were carried out using neuroblastoma SH‐SY5Y cells, which are commonly employed as neuronal models.^[^ ^27^, ^41^, ^42^ ^]^ The main advantage of employing SH‐SY5Y cells is their easy attachment and rapid growth on multiple surfaces and their simple and fast differentiation into neuron‐like morphology, offering insights into the behavior of mature neurons.^[^ ^27^ ^]^ Thus, these cells are an excellent model in tissue engineering to test materials as substrates and assess their maturity and differentiation effect by examining factors like morphology TUBB3, as well as cell coverage and viability. Consequently, data derived from studies using SH‐SY5Y cells can be extrapolated to mature neurons in a more cost‐effective and time‐efficient manner^[^ ^27^, ^41^, ^42^ ^]^

Both PIM and LK hydrogels were confirmed to be biocompatible by LDH cytotoxicity assay. (Figure S8, Supporting Information). Given the bioinert nature of PVA, which disfavors cell attachment, it is evident that CNTs concentration had a minimum threshold after which cells start to attach, dictating how much of the CNTs is exposed for cell interaction. In this line, we observed that cells require at least 60% w/w of CNTs (PIM60) to attach and grow (Figure S9, Supporting Information). Therefore, the effect of CNTs on cell attachment was studied employing triplicates of PIM60, PIM70, and PIM75. After 10 days of incubation with neuroblastoma cells, no significant difference between PIM60 versus PIM70 was observed, both measuring around 5% cell coverage however, cell coverage increases to 11% as the CNT concentration pushes the 75% w/w limit (Figure S9, Supporting Information).

In order to enhance the onset interaction between CNTs and cells, various coatings were employed (Figure S10, Supporting Information). Once chosen, PDL coating protocol was performed in all gels to enhance the onset interaction between the cells and the matrix, which revealed threshold‐dependent behavior when analyzing cell coverage and maturity (Figure S10, Supporting Information).

#### 3.4.1 SH‐SY5Y Incubated with PIM Hydrogels Reveals First Threshold‐Dependent Cell Coverage

As discussed before, cell attachment was proven to be dependent on the CNT concentration staring from 60% w/w of CNT without any coating, thus laying out the first threshold effecting cell coverage. This threshold describes the minimum amount of CNT needed to override the interaction of CNT‐matrix and promote the free interaction of CNT with cells.

**Figure 3:**
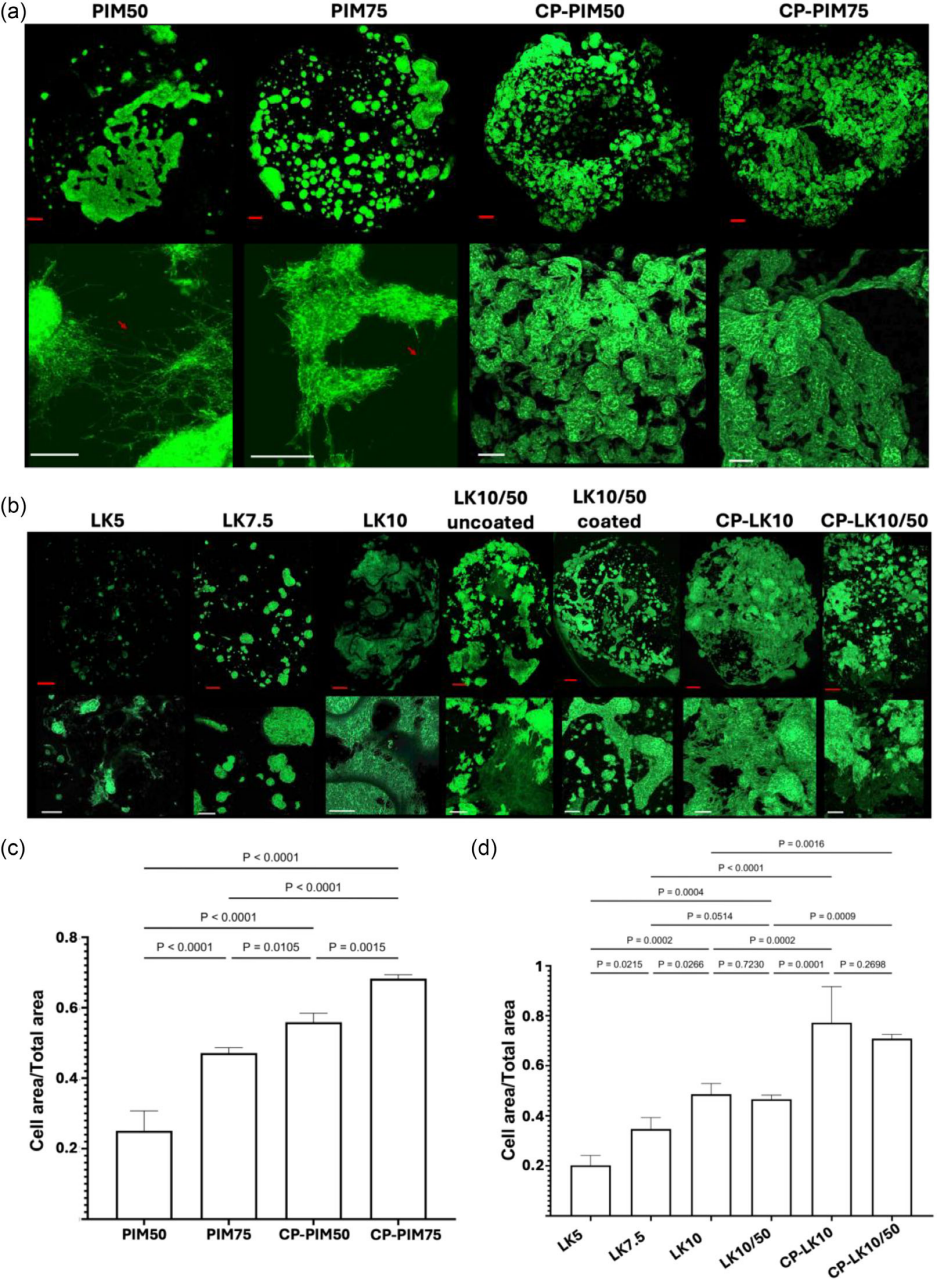
Confocal f‐actin immunofluorescence images for both PIM and LK hydrogels. a) Confocal images of PIM and CP‐PIM hydrogels incubated for 10 days with SH‐SY5Y neuroblastoma cells. b) Confocal images of LK and CP‐LK hydrogels incubated with SH‐SY5Y cells for 10 days. c,d) Cell coverage quantification by image j software. Data presented is a mean of *n* = 3 (1n = triplicates of each sample for a total of nine replicates of each sample). All images were taken at a 10x magnification, 0.5x Zoom. Bar graphs represent mean ±SD. One‐way ANOVA model was used for statistical analysis. Red scale bar is 500 μm, white scale bar is 200 μm.

Following the previous results, two PDL‐coated samples were isolated, namely PIM50; being the lower limit of CNT concentration whereby without coating no cell attachment was seen, and PIM75; upper CNT concentration limit. Morphologically, Cell processes were seen to reach 200 μm in length in both PIM50 and PIM75, due to the inherent large amount of CNTs in both materials (**Figure** **3**a). Measuring the cell coverage on top of the seeded side of the hydrogel, cells on PIM50 covered 29.23% of the hydrogel compared to 48.62% cell coverage for PIM75 (Figure 3a,c). Thus, the same CNT effect observed previously without coating (Figure S9, Supporting Information) was maintained, where increased CNT concentration corresponded to higher cell coverage. It is noteworthy that the difference in cell coverage between PIM50 and PIM75 does not allow the assessment of TUBB3 expression and therefore the assessment of CNT effect on neuron maturity. In this sense, PIM75 will always show higher TUBB3 signal even if maturity in both is the same, and even when reporting tubulin/actin signal, this effect cannot be mathematically corrected for.

#### 3.4.2 SH‐SY5Y Incubated with LK Hydrogels Reveals Second Threshold‐Dependent Cell Coverage

The mechanism of CNT–PVA interaction is largely outlined by the polarity of functional groups on PVA, electrostatic interactions, and hydrogen bonding, this is known to yield PVA wrapping around the CNT aggregations or individual CNTs.^[^ ^43^ ^]^ This mechanism of interaction greatly relates to the interpretation of our in vitro analysis with LK hydrogels. In our study it is expected that the higher the PVA concentration, the higher the degree of interaction between PVA and CNT, which in turn signifies a lower exposure of CNT's surface to interact with cells and vise versa. In other words, if there is more PVA polymers wrapping around CNTs, those CNTs will not be available for CNT–Cell interaction. In extension to the previous reasoning and examining the cell coverage, LK5 with lower PVA concentration is expected to interact with cells the most, followed by LK7.5, then LK10 containing the highest amount of PVA and hence lowering CNT exposure due to increased CNT wrapping and therefore, supposedly, reaching a similar conclusion about CNT effect as described in the previous subsection. Contrary to these expectations, LK5 with YM of 30.24 kPa showed around 20% cell coverage this further increases as we increase PVA's concentration and, by extension YM (Figure 3b,d). This increase reaches around 35% cell coverage for LK7.5 with YM of 72.59 kPa and around 50% cell coverage for LK10 with YM of 207.33 kPa. The previous findings suggest another threshold‐dependent behavior, that of YM, below which YM effect dominates in the hydrogel understudy regardless of CNT interaction with the cells. With PIM hydrogels, the YM was well above 0.2 MPa which did not allow YM effect to dominate even when comparing PIM50 with YM of 1.5 MPa versus PIM75 with YM of 66 MPa a 4x folds difference from that of PIM50.

Interestingly, increasing the CNT concentration of the highest‐performing hydrogel LK10 from around 9% w/w to 50% w/w (LK10/50) shows a plateau of 50% cell coverage regardless of the presence of a PDL coating or not (Figure 3b,d). Therefore, satisfying the previous observation whereby YM dominates CNTs effect.

#### 3.4.3 Effect of Controlled Porosity

The hypothesis behind the incorporation of pores of at least 100 μm arises from the reasoning that cells would penetrate deeper, be exposed to better nutrients/residue flow, and liberate their own ECM/secretions more easily, which, if contained within a confined environment, could exert external physical stress on the cells and eventually alter their morphology or their ability to divide or grow^[^ ^35^, ^36^, ^44^ ^]^

**Figure 4:**
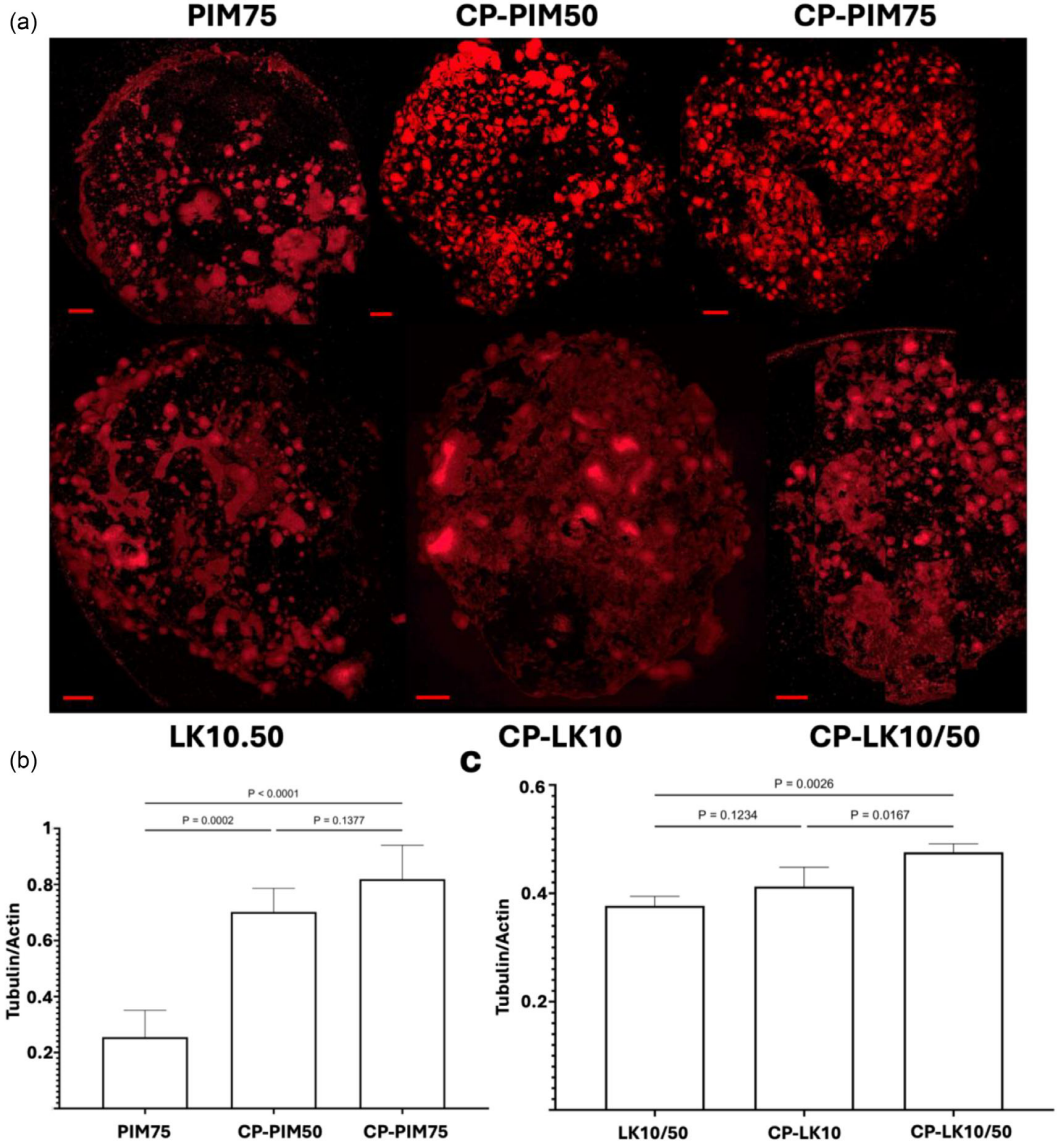
Confocal immunofluorescence images of TUBB3 marker. a) Confocal images of TUBB3 immunofluorescence in highest performing and highest CNT‐containing hydrogels, namely, PIM75 and LK10/50 both compared to the controlled porosity form of the hydrogels, that is, CP‐PIM75 and CP‐LK10/50. Additionally, to check for TUBB3 expression pertaining to a synergistic effect between CNT concentration and porosity effect, CP‐PIM50 and CP‐LK10 were analyzed too. The statistical data analysis for the expression of TUBB3 was carried out by ImageJ software and reported as Tubulin/actin intensity for b) PIM hydrogels and c) LK hydrogels. Data is presented as a mean of triplicates of whole gel area. All images were taken at a 10x magnification, 0.5x Zoom. Bar graphs represent mean ±SD. One‐way ANOVA model was used for statistical analysis. Scale bar is 500 μm.

Interestingly, controlled porosity allowed cell coverage to break through established thresholds. PIM75 witnessed an increase from its previous maximum cell coverage of 49% to *c.a* 70% for CP‐PIM75 (Figure 3a,c). The same effect was seen with LK hydrogels whereby, LK10/50 showed an increase in cell coverage from 50% to *c.a* 78% for CP‐LK10/50 breaking the previously seen plateau (Figure 3b,d). On the other side, when analyzing the effect of the amount of CNT, the difference in performance between CP‐LK10 and CP‐LK10/50 samples is not significant (*p* = 0.26), displaying a bigger SD as shown in Figure 3d. This finding suggests that for these crosslinked hydrogels with more than 10% of CNTs (LK10 and CP‐LK10), the effect of the amount of CNTs becomes less relevant with bigger pore size, implying that porosity has a more dominant effect than CNT content. Similarly, LK10 vs LK10/50 showed no difference suggesting that even if the YM is a dominant factor and even if the YM increased (with the introduction of 50% CNT in LK10/50), there is a plateau at 10% of CNT. Interestingly, we note that this behavior contrasts with our observations in PIM hydrogels, where both porosity and CNT content significantly influenced cell coverage (see Figure 3c). Additionally, an increase in TUBB3 expression (biomarker for neuronal progenitors and neural maturation) from 0.25 a.u for PIM75 to 0.84 a.u for CP‐PIM75 and 0.38 a.u for LK10/50 to 0.47 a.u for CP‐LK10/50 indicated a clear link between pore size and maturity (**Figure** **4**). In this sense, increasing the pore size increased cell–cell interaction while maintaining cell–matrix interactions allowing 3D directional expansion of SH‐SY5Y cell aggregates (Figure 3b, **CP‐LK10** and **CP‐LK10/50**).

In contrast, the previously described effect of CNTs was preserved in CP‐PIM hydrogels. CP‐PIM75 exhibited enhanced cell coverage (70% cell coverage) compared to CP‐PIM50 (56% cell coverage), reinforcing the contributory role of CNTs (Figure 3a,c). Interestingly, this trend was absent in CP‐LK hydrogels similar to their pristine LK counterparts thus, demonstrating comparable cell coverage values of 78% and 72% for CP‐LK10 and CP‐LK10/50, respectively (Figure 3b,d). From this perspective, CNT effect was never a dominant factor in LK hydrogels and therefore even when varying the CNTs concentration more than 5x within CP‐LK formulations did not yield discernible differences in cellular response. This implies that intrinsic bioactive cues within a hydrogel matrix could synergistically dominate within a material even in the presence of another. That is, controlled porosity effect alongside CNT effect dominating in PIM and CP‐PIM hydrogels and controlled porosity effect alongside YM effect dominating in LK and CP‐LK hydrogels.

#### 3.4.4 LK Hydrogels Exhibited Cell‐Mediated Matrix Remodeling

As an attempt to measure and analyze 3D cell morphology within a polymeric network and examine the effect of the cells on the gel integrity, SEM analysis was carried out in LK10 hydrogel incubated with SH‐SY5Y cells for 3 days as a representative sample.

**Figure 5:**
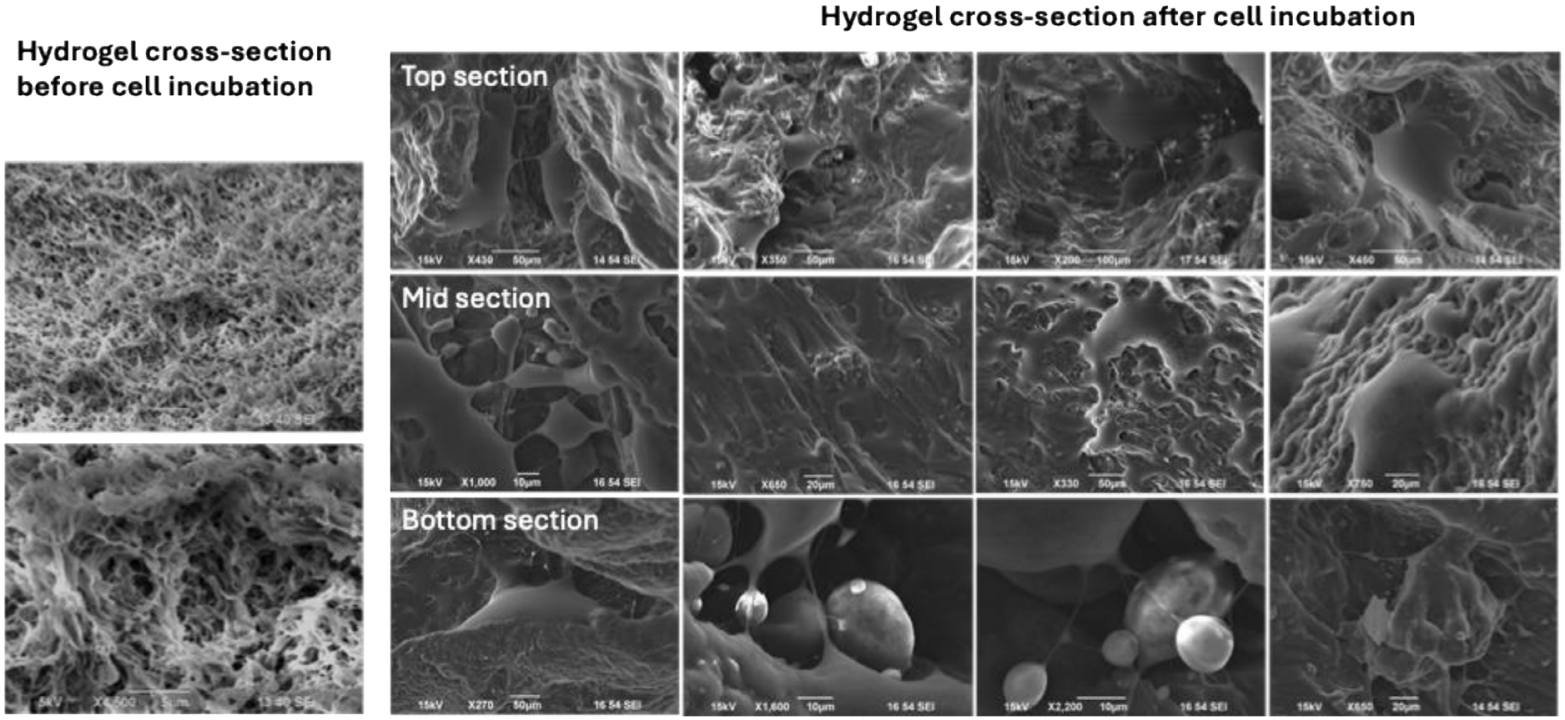
SEM images of LK10 hydrogel incubated with SH‐SY5Y cells. SEM analysis was carried out after 3 days of incubation at different sections of lyophilized LK10 hydrogels. The analysis was carried out by comparing LK10 hydrogel before cell incubation (cross‐section ‐ left) and after (gel interior ‐ right) starting from the top section of the gel to a higher depth.

The resulting SEM images, in turn, revealed that cells effectively penetrated the entire hydrogel proving the efficacy of the intrinsic hydrogel pore network in facilitating cell infiltration. However, the porous hydrogel interior experienced complete remodeling of its structure whereby cells liberated their own secretions and created tunnel‐like structures where cells could also interact with other cells (**Figure** **5**, top section). Additionally, cell processes protruded from the cells, interacting with both the matrix and adjacent cells from all directions while maintaining their 3D morphology, unlike 2D cultures (Figure 5). This effect is reported faintly in literature and mostly for ECM‐based materials, known as cell‐mediated matrix remodeling, which involves the cell's ability to rearrange the ECM and frequently remodel it according to their expansion needs.^[^ ^45^ ^]^ Therefore, LK hydrogel revealed that cell‐mediated matrix remodeling can occur within a synthetic network and accommodate for the external forces exerted by the cells onto the matrix.

## 4 Conclusions

In conclusion, PIM resulted in better polymer compacting, which in turn allowed for more CNTs loading up to 75% w/w compared to 50% when employing low kinetic crosslinked hydrogels (LK). Not only did both hydrogels show a YM in the order of MPa, which is close to native tissue, but also, adopting j‐shaped curve and experiencing fracture‐resistant and deformation‐resistant behavior like the targeted biological tissue.

The bioactivity of CNTs in promoting cellular growth was validated across both hydrogel systems (PIM and LK), critically addressing prevailing concerns regarding CNT cytotoxicity at elevated concentrations. CNT loadings in the range of 100–420 mg mL^−1^ performed similar to synthetic expansion factors, namely, PDL as seen with LK10/50 hydrogel when studied without any treatment.

Upon incubating PIM and LK hydrogels with SH‐SY5Y cells, a threshold‐dependent cell growth was seen, allowing one key factor to dominate a material's behavior in determining cell proliferation and coverage in efforts to create stable tissue‐like structure in a synthetic material. Most notably, when the YM is above a certain threshold, the effect of CNT is dominant thus showing highest cell coverage with increasing CNT concentration. Whereas YM dominates when it is below a certain threshold regardless of CNT effect and interaction with cells. This dual‐mode influence was further modulated by pore architecture, where increased pore size synergistically enhanced cellular infiltration and overcame saturation plateaus in cell coverage, while also upregulating the expression of neuronal maturity markers. Notably, LK hydrogels served as a suitable platform for elucidating cell‐mediated remodeling within a synthetic matrix. These hydrogels supported endogenous ECM‐like remodeling without compromising structural integrity, thereby permitting cell infiltration throughout the 3D scaffold, sustained cell secretion into the matrix, and preservation of native‐like cellular morphology.

Collectively, these findings highlight critical parameters, elastic modulus, CNT loading, and microstructural tuning (pore size control), that must be strategically optimized for the rational design of next‐generation scaffolds aimed at nerve regeneration. This study underscores the potential to provide precise control and prediction of the ability of these hydrogels to accommodate for complete tissue formation within their network and thus, permitting functional nerve restoration.

## Supporting Information

Supporting Information is available from the Wiley Online Library or from the author.

## Conflict of Interest

Materials reported are protected under the processing of patent number PCT/EP2024/071248.

## Author Contributions

The manuscript was written through contributions from all authors.

## Supporting information

Supplementary Material

## Data Availability

The data that support the findings of this study are available from the corresponding author upon reasonable request.
