# Supplementary material for: Carbon Nanotube Hydrogels Reveal Threshold‐Dependent Regulation of Neuroblastoma Cell Growth and Maturation by Mechanical and Chemical Factors
Source: Small Sci. 2025 Sep 18;5(11):2500401. doi: 10.1002/smsc.202500401 (PMC12622437; doi:10.1002/smsc.202500401)
Supplement: Supplementary file 1 — Supplementary Material [file SMSC-5-2500401-s001.pdf]

Carbon Nanotube Hydrogels Reveal Threshold-Dependent Regulation of Neuroblastoma Cell Growth and Maturation by Mechanical and Chemical Factors

Bahaa Daou,[1,2] Maurizio Prato,[1,3,4,]* Sonia Alonso-Martín,[2,5,]* Nuria Alegret.[3,6]*

[1] Center for Cooperative Research in Biomaterials (CICBiomaGUNE), Basque Research and Technology Alliance (BRTA), Donostia/San Sebastián, 20014, Spain

[2] Stem Cells and Aging Group, Bioengineering Area, Biogipuzkoa Health Research Institute, 20014 Donostia/San Sebastián, Spain

[3] Ikerbasque, Basque Foundation for Science, Bilbao, 48013, Spain

[4] Department of Chemical and Pharmaceutical Sciences, Universitá Degli Studi di Trieste, Trieste, 34127, Italy

[5] CIBERNED, ISCIII (CIBER de Enfermedades Neurodegenerativas, Instituto de Salud Carlos III), 28031 Madrid, Spain

[6] Cardiac Diseases Group, Systemic Diseases Area, Biogipuzkoa Health Research Institute, 20014 Donostia/San Sebastián, Spain

Supporting Material

1. Results and Discussion

Adding to the structural analysis of PIM hydrogels, TGA estimation of CNT percentage in the final material is reported in **Figure S1a**. Even at higher concentrations, a good amount of CNTs is present in the final material compared to the theoretical percentages, proving the efficiency of the preparation process. To simplify the forthcoming results, we have chosen 3 PIM hydrogels to extensively evaluate the physicochemical properties as reference models for all the multiple PVA:CNT ratios possible: PIM20 lower end of CNT concentration at 20% w/w, PIM50 middle end at 50% w/w and PIM75 upper end at 75% w/w.

FTIR-ATR spectroscopy was used to confirm the chemical structure and evaluate possible interactions in the supramolecular arrangement of the hydrogels. At low CNT concentration (PIM20) the typical spectrum of pure PVA is shown, with -OH stretching at around 3250 cm^{-1} and -CH stretching at 2900 cm^{-1}; remnants of acetate from PVA's synthesis polymerization reaction is detected at ~1650 cm^{-1} (**Figure S1b**). With the increase of CNT concentration (PIM50), the vibrations are masked gradually. To understand the nature of the interaction between PVA and CNT, we examine two samples PVA-pCNT mix; comprised of a simple blend of PVA and pristine CNTs mixed in powder form (20% w/w), and PIM20; formed via PVA/CNT dispersion and followed by phase inversion. In the first case and although CNT is present at the same concentration as PIM20, CNT masks the typical vibrations of PVA and results in new vibrations most prominently around 1950 cm^{-1}, 2050 cm^{-1}, and 2200 cm^{-1} which can be attributed to various defects on the surface of CNT. Examining the same sample, CNT causes -OH stretching to shift to around 3600 cm^{-1} in addition to masking the entire fingerprint region which normally appears in pure PVA or even in PIM20. Such behavior is not seen in PIM hydrogels suggesting that the PVA spectral domination arises from the wrapping of CNT by the PVA chains via van der Waals forces, and/or π-π interactions.

It is known that the lesion-site brings many post-traumatic inflammatory events, such as slight decrease in pH, hemorrhage and the infiltration of fibroblasts[1–3]. These events can change the chemistry of hydrogels and render them weak leading to increased swelling and eventually degrading in the host organism. Hence, one of the most important characteristics when engineering CNT-based hydrogels is their stability in physiological medium, and the swelling-resistance properties. Indeed, phase inversion generally resulted in a swelling resistant behavior suggesting tight stacking between PVA chains as the polymer rich phase separates and solidifies (**Figure S1c**). Moreover, the hydrogels reached volume and weight equilibrium after only 4 hours at 37 $^{\circ}$C in physiological conditions. At lower CNT concentration, the hydrogels are less resistant to swelling with around 450% and 300% swelling by weight for PIM20 and PIM50, respectively, compared to ca. 200% for PIM75 owing to the hydrophobic nature of CNTs (**Figure S1c**). On the other hand, CNTs contribute to the structural stability, *i.e.,* swelling by volume, of the hydrogels (**Figure S1d**), hereby, resistance to deformation in terms of volume shrinkage and swelling, becomes evident at higher CNT concentration dropping from 100% swelling by volume for PIM20 to *ca.* 40% and *ca.* 30% for PIM50 and PIM75, respectively. Interestingly, applicable for all PIM hydrogels and probably due to the elasticity of PVA, swelling by volume in PBS does not pass beyond that of water at equilibrium, suggesting that the original volume shrinkage, which happens after reaching equilibrium in water followed by freeze-drying (the process of freeze-drying does not preserve completely the structure of the hydrogels), is entirely regained and no further swelling occurs. Moreover, As an estimation to the conductivity behavior of these gels, 4-PP was used to measure sheet resistance in PBS using few mm-thick PIM20, PIM50 and PIM75 hydrogels (**Figure S1e**), and in water for all the PIM hydrogels (**Figure S1f**). The measurement taken for hydrogels prepared in water show that the sheet resistance of all PIM hydrogel up to 70% of CNTs is between 50-60 kΩ/square, while in PIM75 the sheet resistance drops to 25 kΩ/square (**Figure**

S1f). This decrease is directly attributed to CNT's delocalized π-clouds only. However, in PBS, both ionic flow and CNT's delocalized π-clouds along the structure contribute to the decrease in sheet resistance. More prominently, as the CNT percentage increases to 75% (750 Ω/square), the sheet resistance falls more than 3x compared to PIM20 (3,200 Ω/square), signaling direct interaction between the CNTs (**Figure S1e**). This interaction could be a key factor in cell to cell and cell to matrix interaction in biological systems. It is worth mentioning that resistivity in 3D systems is largely attributed to the dielectric polymer used, geometrical resistivity (non-linear flow of charge carriers), void/pores (less contact between the conductive additives, *i.e.*, CNTs) and most notably the random distribution of CNTs which makes them align in various planes with respect to the measuring probes.[4]

Furthermore, PIM hydrogels show porosity of around 90% regardless of CNTs concentration for both PIM20 and PIM50, while a decrease to ca. 60% for PIM75 was observed (**Figure S1g**). With liquid displacement method and due to the hydrophilic nature of PVA, some water lingers on the surface of the hydrogel at the time of measurement, which yields higher values with an estimated error close to 10%.

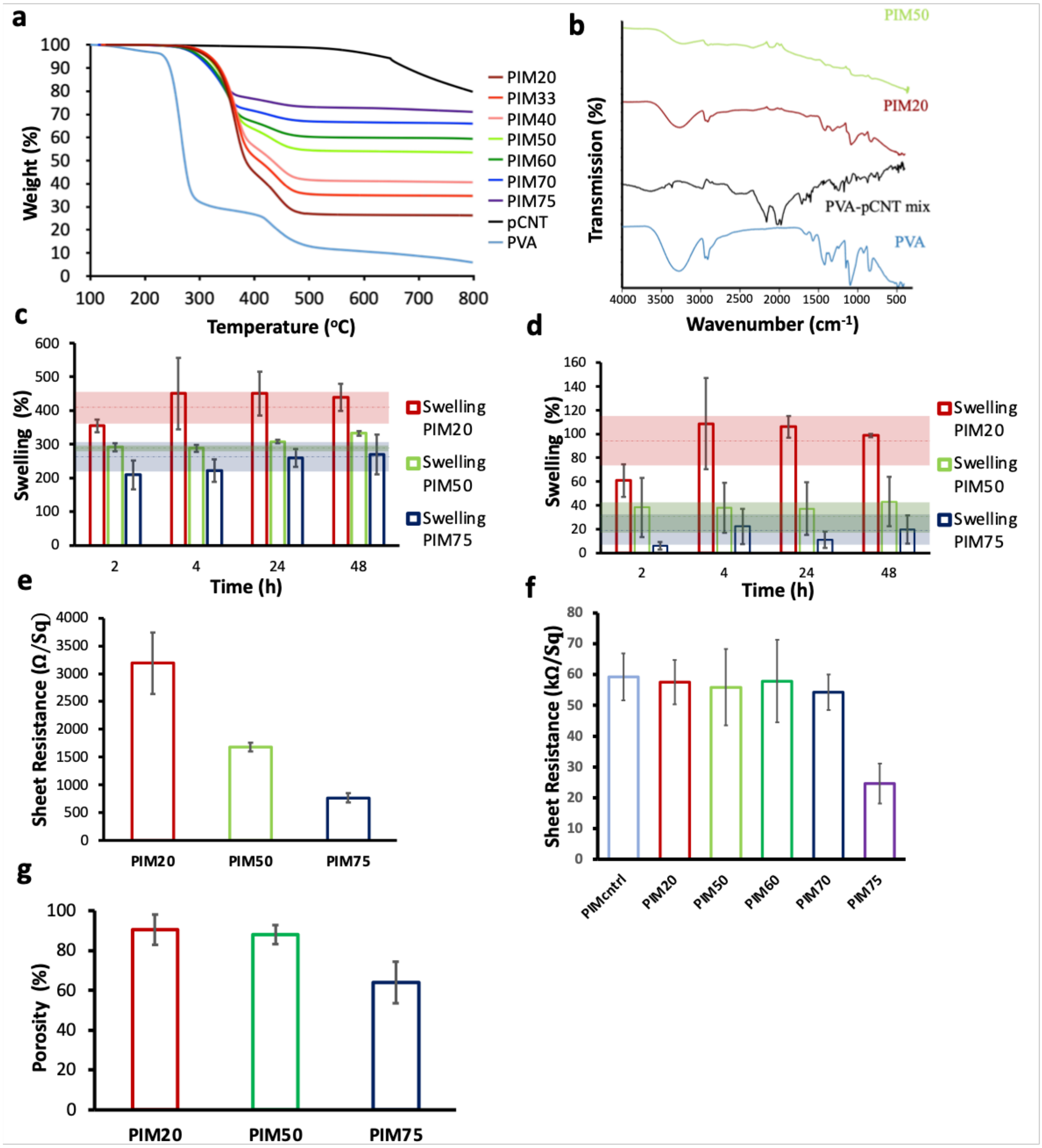

Figure S1. Characterization of PIM Hydrogels. a) Thermalgravimetric analysis (TGA) of all PIM hydrogels at different CNT concentration. b) Structural analysis by Fourier-transform infrared spectroscopy (FTIR). c) Swelling by weight in physiological conditions at 37 ºC, highlighted bar and dashed line represent the mean swelling percentage (initial swelling) by weight ± SD of gels before freeze-drying. d) Swelling by volume in physiological conditions at 37 ºC, highlighted bar and dashed line represent the mean swelling percentage (initial swelling) by volume ± SD of gels before freeze-drying. e) Sheet resistance values of PIM hydrogels using 4-point probe in PBS and f) in water. g) Porosity of PIM hydrogels using liquid displacement method. PIM numbers indicate CNTs %. n=3. Bar graphs represent mean ±SD.

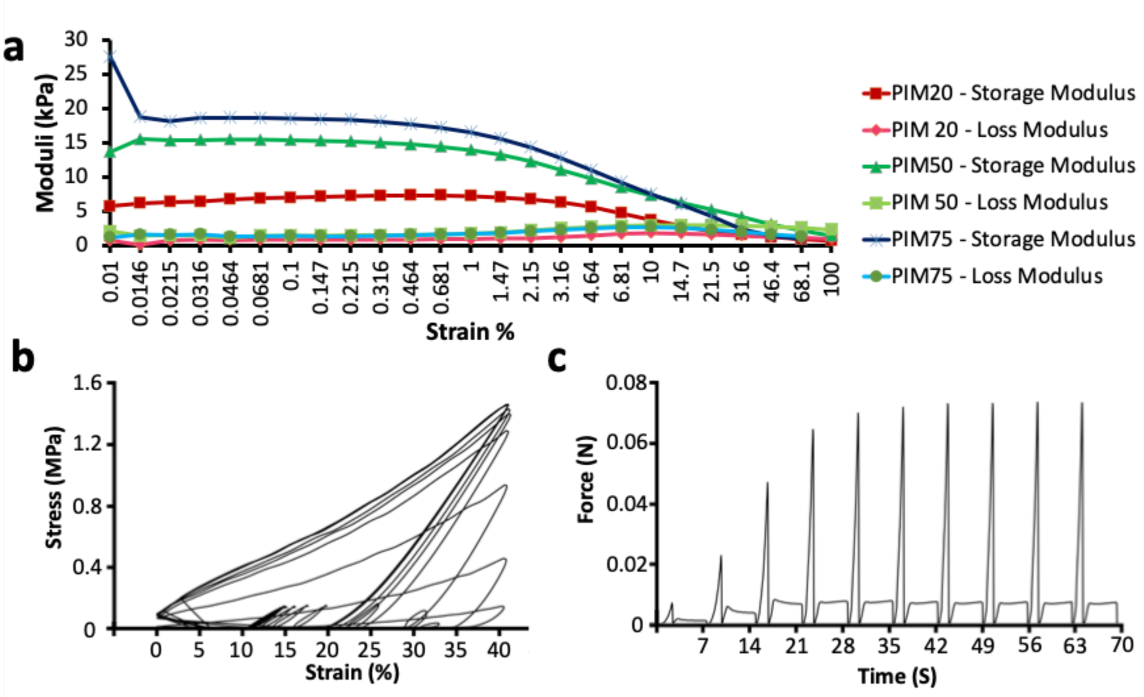

Figure S2. Mechanical and rheological properties of PIM hydrogels. a) Amplitude sweep plot for PIM hydrogels at a constant frequency of 10 rad/s. b) Cyclic stress-strain curve from uniaxial compression test performed using universal testing machine of PIM50 taken as a median for PIM hydrogels. c) Force-time curve illustrating the cyclic pattern carried out for PIM50.

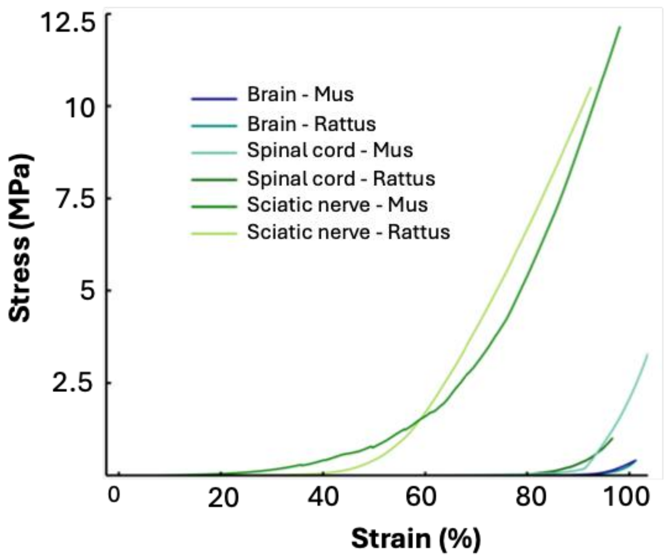

Rattus	Young's Modulus (kPa)	SD (kPa)
Brain	2.05	0.0081
Spinal cord	11.46	0.65
Sciatic nerve	538.33	97.91

Mus musculus	Young's Modulus (kPa)	SD (kPa)
Brain	1.98	0.14
Spinal cord	11.44	1.71
Sciatic nerve	515.33	55.55

Figure S3. Mechanical properties of various nerve tissues. a) Uniaxial compression test of various nerve tissues of Sprague-Dawley® rats (*Rattus*) vs. C57BL/6J *Mus musculus* (Mus). and the corresponding young's moduli for both species. n=3 ± SD.

Scanning electron microscopy was used to analyze pore size distribution.[5–7] SEM images show a general uniform structure in all PIM hydrogels (**Figure S4a**). In particular, they have bigger and more randomly distributed porous network on its surface compared to a more homogenous network and smaller mean pore size on the side in contact with the glass substrate. Surprisingly, high CNT concentration did not influence negatively the pore size distribution. On the contrary, the mean pore size was in the range of 10 µm to 17 µm for PIM50 and PIM20 respectively and increasing to c.a. 30 µm for PIM75 (**Figure S4b-d**). This could be attributed to the tight compacting of PVA as discussed earlier, which wraps around the CNTs stabilizing them. This effect was seen starting from 50% w/w of CNT (PIM50), whereby, large CNT/PVA aggregates formed creating well defined pores in between said aggregates. Eventually, these aggregates would be expected to either form pathways for cells to infiltrate the hydrogel or simply to grow on top of them. It is noteworthy that, PIM20 showed directionality dependence of the PVA/CNTs fibers within the hydrogel's network parallel to the drag force upon layer-by-layer formation, due to the pull and drag forces applied when synthesizing these gels. In summary, PVA has proven to be a good candidate for loading very high concentrations of CNTs without jeopardizing the pore size and subsequently the cells' ability to infiltrate and proliferate within a 3D network.

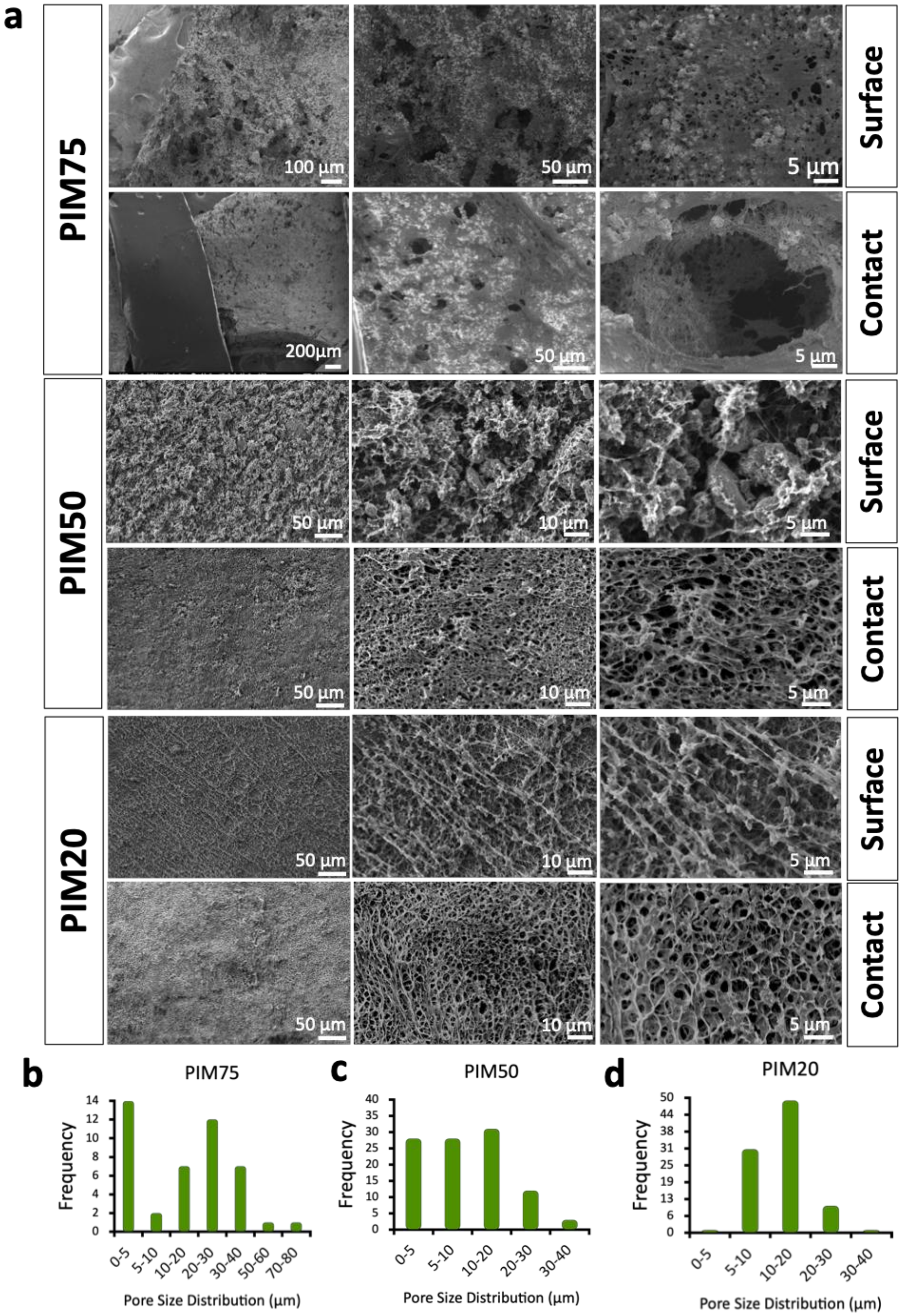

Figure S4. SEM images for PIM hydrogels and pore size analysis. a) Representative images for the surface topography analysis of PIM hydrogels through SEM after lyophilization. The images shown compares surface pore size distribution (denoted by surface) and the side in contact with the glass slide during synthesis (denoted by contact). b-d) Pore size distribution for each PIM hydrogel (n=50 pores for PIM75 and n=100 pores for PIM20, PIM50 from 3 different zones of different hydrogels).

Following the same methodology of physicochemical characterization, TGA analysis was used to estimate the experimental CNTs concentration in LK hydrogels, which measured 12.46%, 14.60%, 13.78% and 56.50% for LK5, 7.5, 10 and 10/50, respectively similar to the theoretical values proving the efficiency of the synthesis process (**Figure S5b**).

FTIR-ATR analysis was employed to evaluate the crosslinking mechanism within the hydrogels (**Figure S5c**). The typical 4ABA bands were marked by sharp signals at 3,500 cm^{-1} for -NH stretching and a broad band centered at 2,700 cm^{-1} corresponding to -COOH. The lyophilized crosslinked hydrogel resulted a simple overlap of both the PVA and the 4ABA spectra. As expected, the more we increased the concentration of 4ABA, the stronger the 4ABA bands in the hydrogel spectrum (**Figure S5c**). This suggests that the nature of crosslinking is based on hydrogen bonds formed between -amino and -carboxylic groups of the crosslinker and the -hydroxy and -acetate remnants groups on the PVA.

The swelling behavior of the hydrogels was also measured by weight and volume (**Figure S5d**). All the scaffolds reached equilibrium in PBS at 37 ºC after 2 hours and maintained their swollen weight at ca. 1000%, 750%, 600% and 500% for LK5, LK7.5, LK10 and LK10/50, respectively. With the increase of PVA's concentration the hydrogels become more swelling-resistant, likely due to higher degree of crosslinking, which further tightens the distance between the PVA chains.

LK hydrogels, as with PIM hydrogels, experience a shrinkage after lyophilization. Measuring the swelling by volume behavior of LK hydrogels, revealed that all hydrogels recover their original volume (**Figure S5e – highlighted bars)** without further swelling. To this end, they reach equilibrium after 24 hours, measuring an average of 37%, 22% and 25% swelling by volume for LK5, LK7.5, and LK10, respectively (**Figure S5e**). As expected for LK10/50, swelling by volume was

practically negligible of around 5%, due to its high CNT content and superior mechanical properties that resisted shrinkage upon lyophilization and, therefore, no major swelling was seen.

Porosity of LK hydrogels at high concentration was not affected, again owing to the interaction of PVA and CNT and the subsequent compacting within the hydrogels network. To this end, the average porosity calculated by liquid displacement method was 74%, 67%, 82%, and 60% for LK5, LK7.5, LK10, and LK10/50 respectively. (**Figure S5f**)

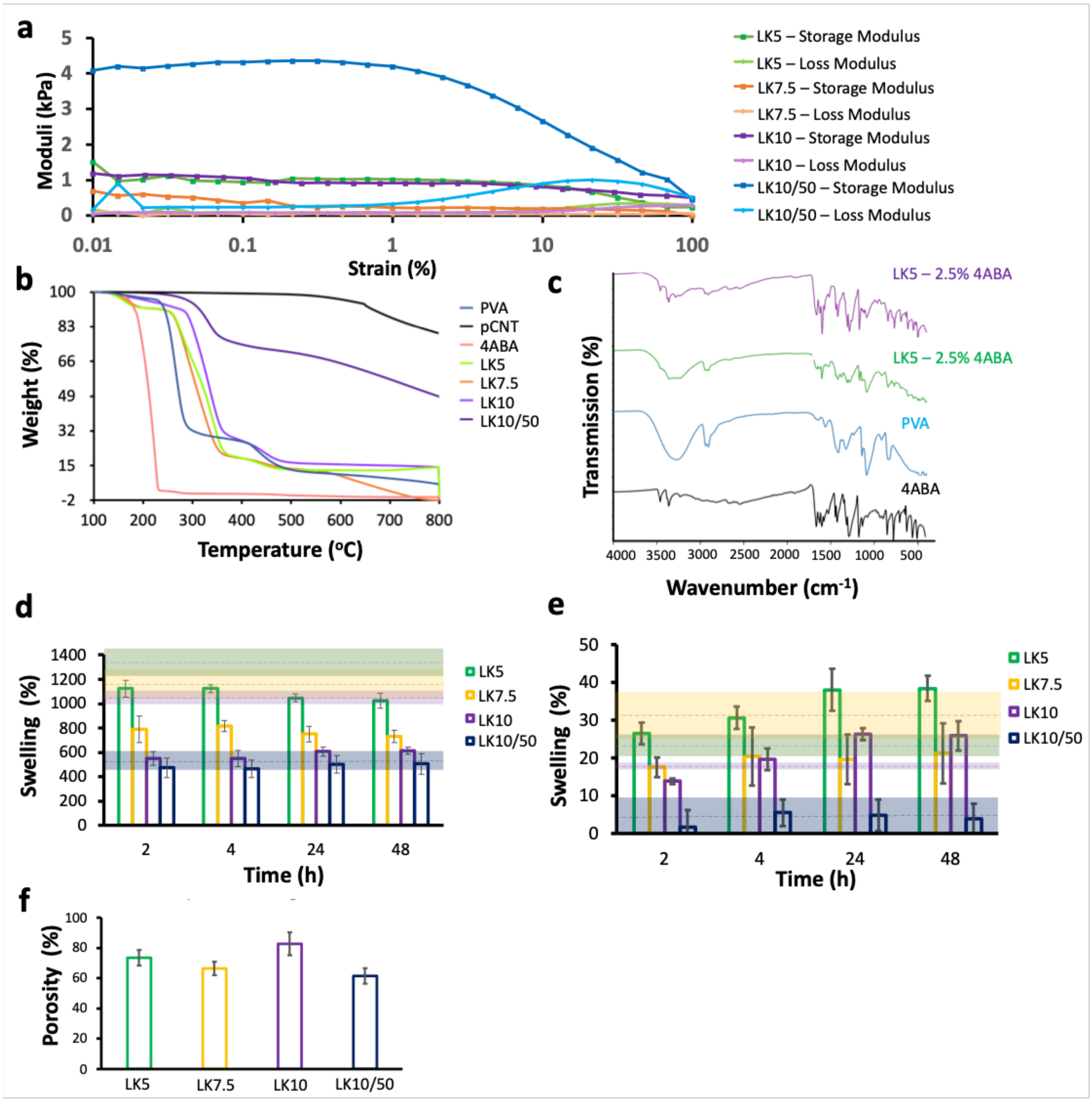

Figure S5. Mechanical properties of LK hydrogels. a) Amplitude sweep plot for LK hydrogels at a constant frequency of 10 rad/s. b) Thermal gravimetric (TGA) analysis of all LK hydrogels at different PVA concentration. c) Fourier-transform infrared spectroscopy (FTIR) showing IR spectra for 4ABA, PVA, and LK hydrogel at two different 4ABA concentrations. d) Swelling by weight in physiological conditions at 37 ºC, highlighted bar and dashed line represent the mean swelling percentage (initial swelling) by weight ± SD of gels before freeze-drying. e) Swelling by volume in physiological conditions at 37 ºC, highlighted bar and dashed line represent the mean swelling percentage (initial swelling) by volume ± SD of gels before freeze-drying. f) Porosity of LK hydrogels calculated using liquid displacement method. n=3. Bar graphs represent mean ±SD.

SEM analysis for LK hydrogels showed similar pore size range as PIM hydrogels. Indeed, this further confirms the suggestion that polymer compacting, and nature of PVA-CNT interaction determines the topographical properties of these hydrogels (**Figure S6**). Most importantly, having similar pore size distribution in both hydrogels avoids having variables that would otherwise affect the in vitro analysis. To this end, pores were seen in the range of 5-20 μm, a deeper analysis revealed that the average pore size for LK hydrogels measured 11.75 ± 8,22 μm, 11.21 ± 6.85 μm, 10.51 ± 7.36 μm for LK5, LK7.5, and LK10, respectively (**Figure S6b-d**). Surprisingly, increasing CNT concentration to 50% CNT (LK10/50), the average pore size increased 4x to *c.a* 40.69 μm yielding a wider pore size distribution similar to the effect seen with PIM75 hydrogel. (**Figure S6e**).

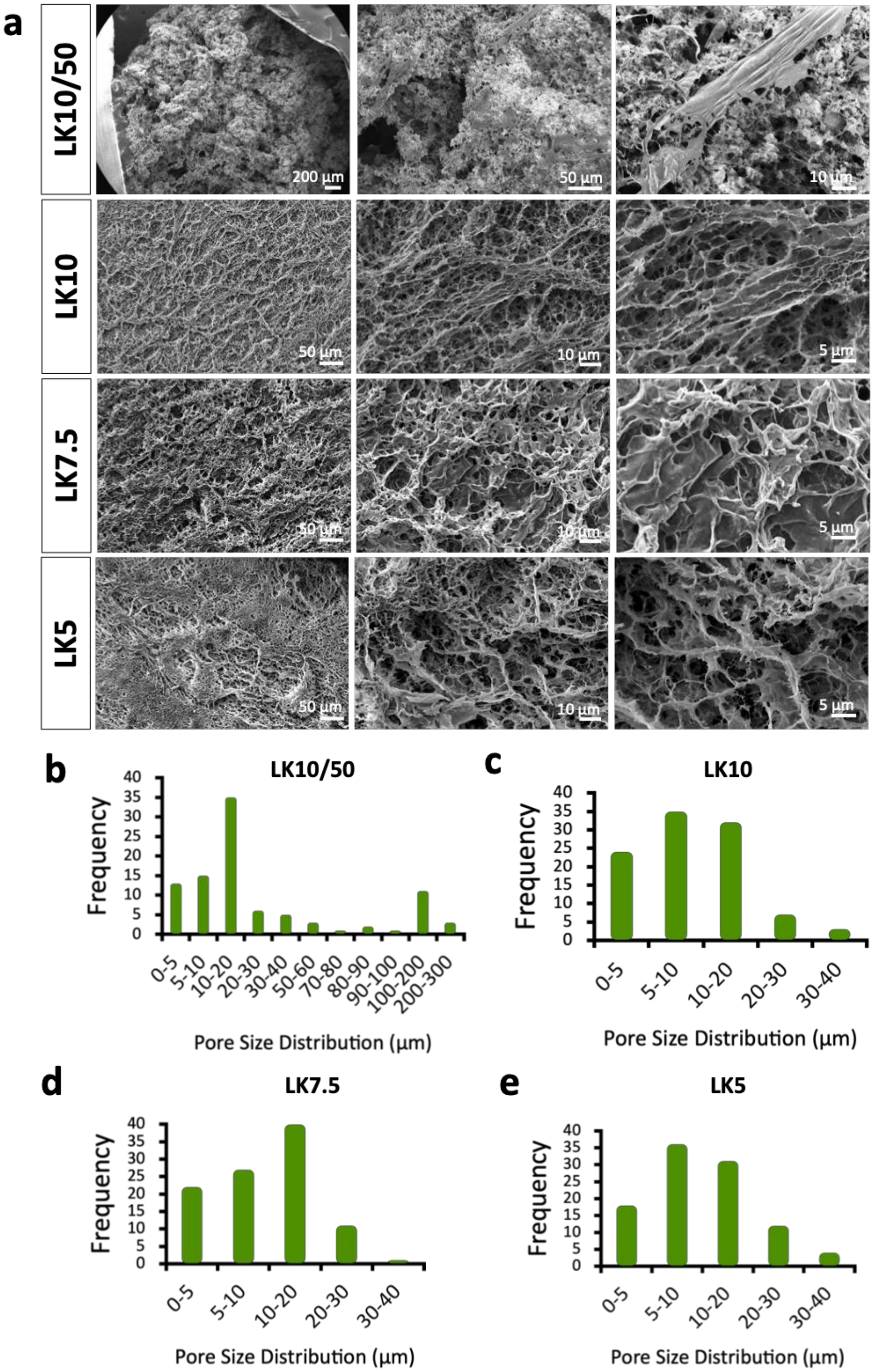

Figure S6. SEM images for LK hydrogels and pore size analysis. a) Representative images of surface topography analysis of LK hydrogels through SEM after lyophilization and (b – e) their corresponding pore size distribution. n=100 pores from 3 different zones of different samples.

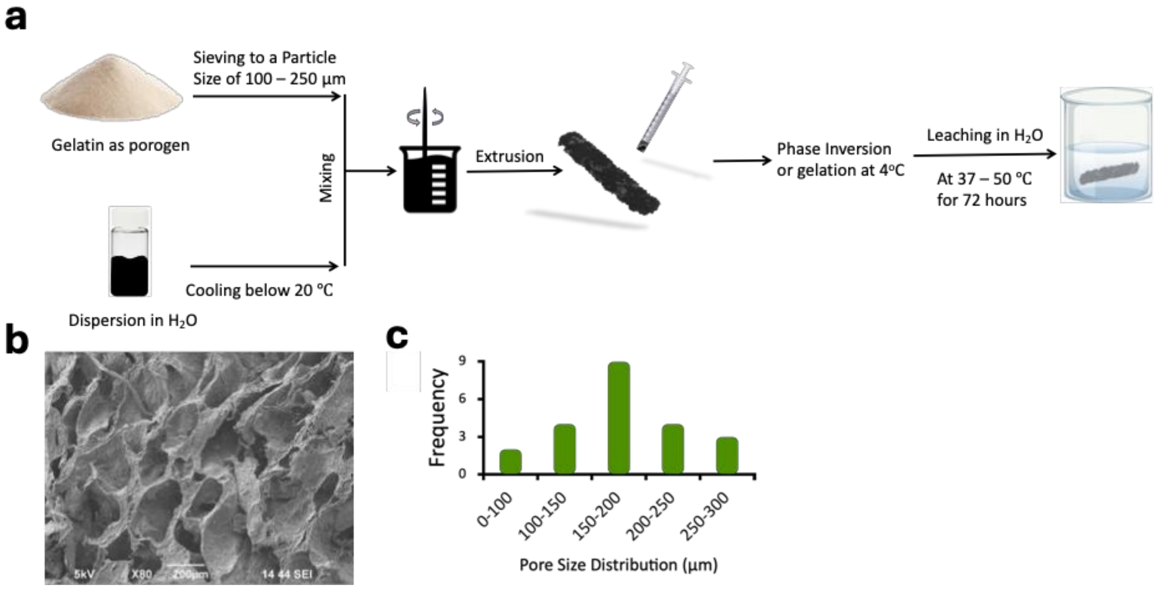

Figure S7. Controlled pore size optimization. a) Schematic representation of the controlled porosity process via porogen leaching method with sieved gelatin particles as porogen, followed by either phase inversion to create CP-PIMs or gelation at 4 ºC to create CP-LKs. b) Representative SEM image for CP-PIM50 taken as a median sample. c) Pore size distribution of CP-PIM50. n=20 pores.

General *In vitro* analysis for the assessment of initial cell attachment, growth and tissue formation using neuroblastoma SH-SY5Y cells

As expected, both PIM and LK hydrogels were shown to be biocompatible as reflected by the LDH cytotoxicity assay (**Figure S8**). To assess the general behavior of PVA/CNT hydrogels and their ability to support 3D tissue formation, PIM hydrogels were used given that both LK and PIM hydrogels have similar base formula. Interestingly, without any further treatment cell attachment was seen starting from 60% w/w of CNTs (PIM60) (**Figure S9**).

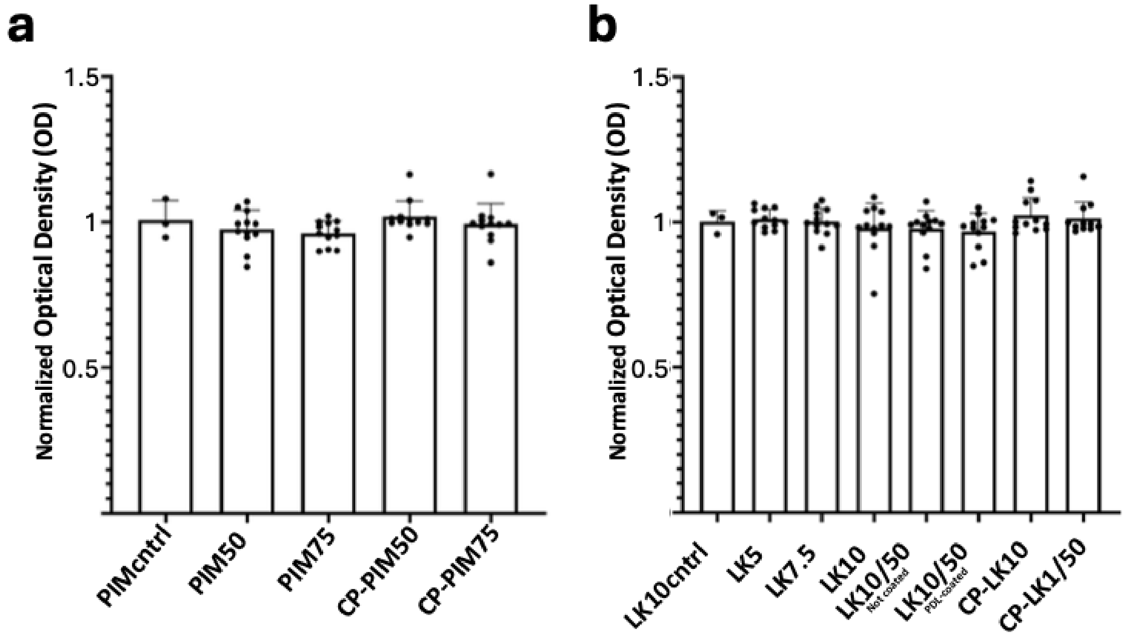

Figure S8. Cytotoxicity analysis for PIM and LK hydrogels. a) Lactose dehydrogenase cytotoxicity assay for PIM and CP-PIM hydrogels coated with PDL and incubated for 10 days with SH-SY5Y cells. Similarly, b) Lactose dehydrogenase cytotoxicity assay for LK and CP-LK hydrogels coated with PDL and incubated for 10 days with SH-SY5Y. n=3 ± SD, each N has 3 repetitions to a total of 9 repetitions of 3 different batches for each sample with the exception of both controls of PIM and LK due to exclusion from further repetitions.

With the aim of allowing full tissue formation on top of PIM and LK hydrogels, the focus is shifted towards increasing the onset interaction between the cells and the matrix (CNTs). Various reported coatings in 2D cell cultures were adapted and modified to fit the 3D hydrogels understudy. Most prominently, HA, Polydopamine (PDA), and PDL and a mixture of those thereof were tested (**Figure S10** and **Figure S9**). In summary, PDL coating alone effectively increased cell attachment and proliferation, allowing morphologically neuron-like maturation with the formation of axon-like processes, reaching a length of >70 μm (**Figure S10a,d**). Contrary to what has been

reported in literature [8], 3D coating with polydopamine showed increased toxicity probably due to the liberation of PDA -micro and nanoparticles with visible aggregations in the supernatant. (**Figure S10b-d**) To avoid bias in the measurements due to this phenomenon, LDH cytotoxicity assay for PDA-containing hydrogels was repeated preceded by filtering the supernatant to remove particles > 0.2 µm and thus decreasing interference with the absorbance at 492 nm. The toxicity effect of PDA was still clear in the LDH results even after filtering, which prompted its dismissal due to its cytotoxicity. On the other hand, HA coating did not show any signs of successful cell attachment but without inducing any cytotoxicity (**Figure S10d**).

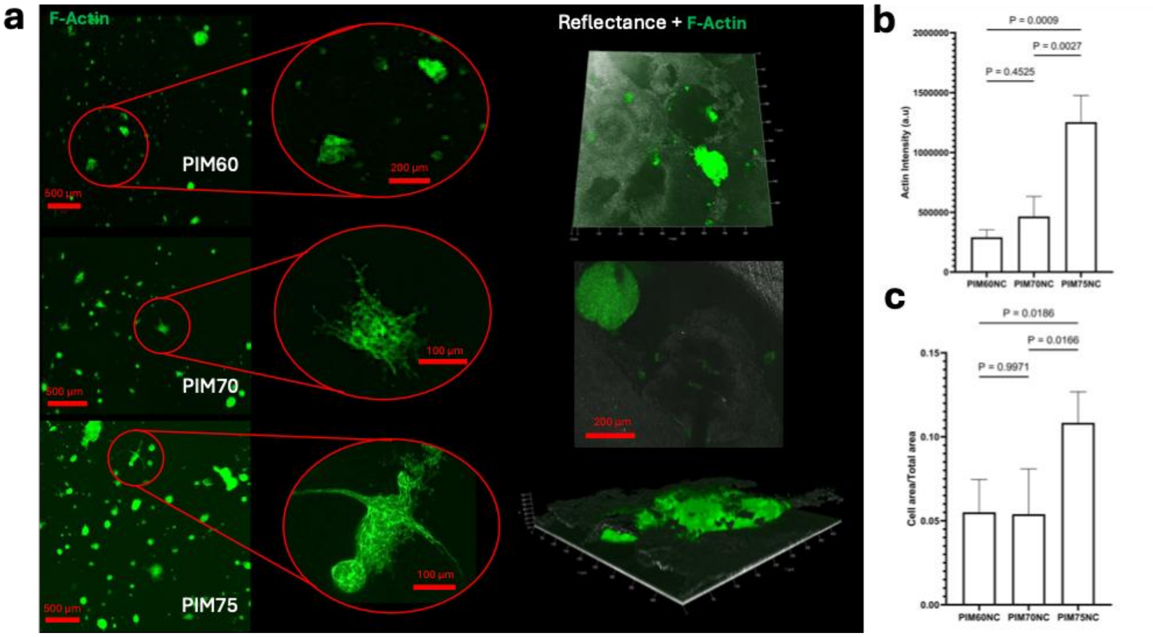

Figure S9. Evaluation of cell attachment and initial cell coverage after 10 days of incubation without any treatment. a) Confocal Images of SH-SY5Y neuroblastoma cells cultured for 10 days on top of untreated PIM hydrogels with different CNTs concentration. b) f-actin intensity measured using phalloidin-Alexa 488 immunostaining with respect to the total area of the hydrogel. c) Cell coverage measured using ImageJ software calculating cell area against total hydrogel area. Triplicates of hydrogels were used. All images were taken at a 10x magnification, 0.5x Zoom. Bar graphs represent mean ±SD. one-way ANOVA test was used for the statistical analysis.

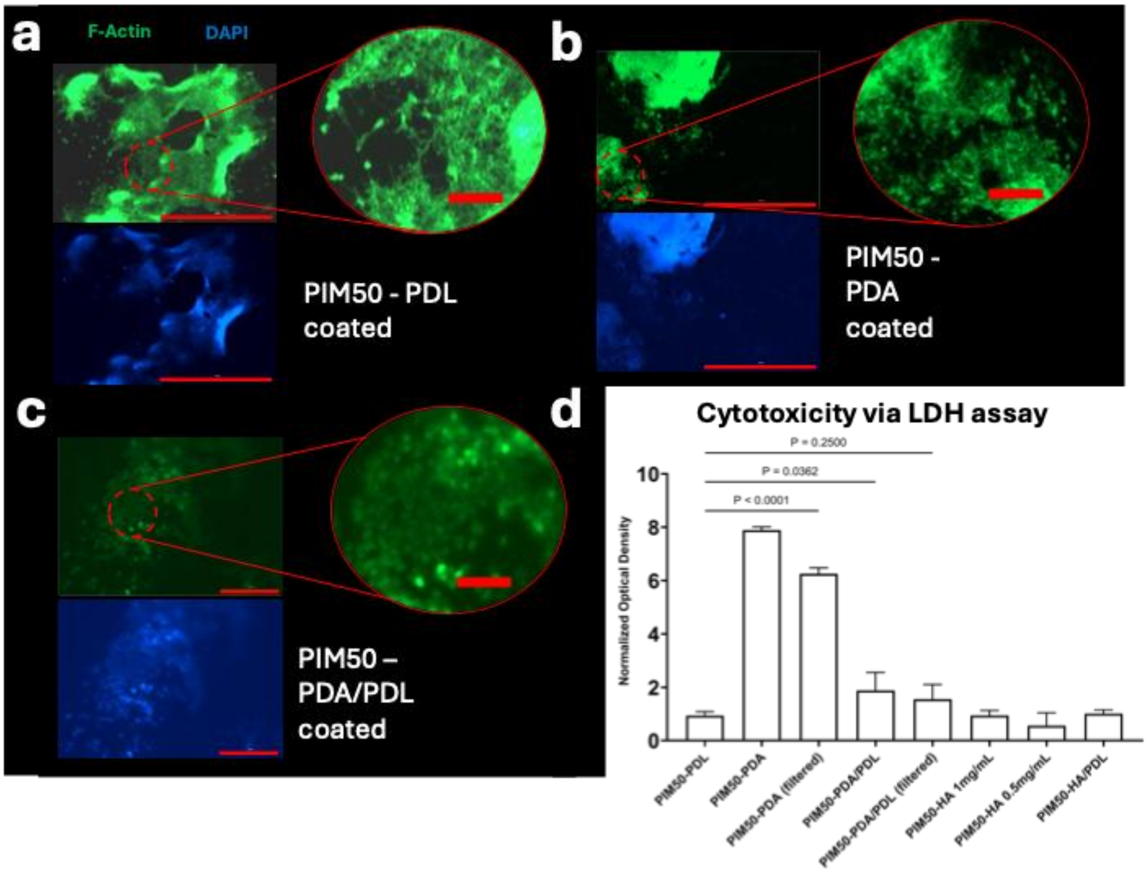

Figure S10. Cell attachment analysis with different coatings adapted to 3D matrices. a) Confocal images of SH-SY5Y neuroblastoma cells cultured for 3 days on top of PIM50 hydrogels with PDL coating, b) PDA coating, and c) PDA/PDL coating. scale bar 1 mm, scale bar of the zoomed images is 100 μm. d) normalized Lactose dehydrogenase (LDH) cytotoxicity assay. Triplicates of hydrogels were used. All images were taken at a 10x magnification, 0.5x Zoom. Bar graphs represent mean ±SD. one-way ANOVA test was used for the statistical analysis.

References:

(1) Holl, D.; Hau, W. F.; Julien, A.; Banitalebi, S.; Kalkitsas, J.; Savant, S.; Llorens-Bobadilla, E.; Herault, Y.; Pavlovic, G.; Amiry-Moghaddam, M.; Dias, D. O.; Göritz, C. Distinct Origin and Region-Dependent Contribution of Stromal Fibroblasts to Fibrosis Following Traumatic Injury in Mice. *Nature Neuroscience 2024 27:7* **2024**, *27* (7), 1285–1298. https://doi.org/10.1038/s41593-024-01678-4.

(2) Ayazi, M.; Zivkovic, S.; Hammel, G.; Stefanovic, B.; Ren, Y. Fibrotic Scar in CNS Injuries: From the Cellular Origins of Fibroblasts to the Molecular Processes of Fibrotic Scar Formation. *Cells* **2022**, *11* (15), 2371. https://doi.org/10.3390/CELLS11152371.

(3) Jin, H.; Liu, Y.; Liu, X.; Khodeiry, M. M.; Lee, J. K.; Lee, R. K. Hematogenous Macrophages Contribute to Fibrotic Scar Formation After Optic Nerve Crush. *Mol Neurobiol* **2022**, *59* (12), 7393. https://doi.org/10.1007/S12035-022-03052-6.

(4) Silva, J.; Ribeiro, S.; Lanceros-Mendez, S.; Simões, R. The Influence of Matrix Mediated Hopping Conductivity, Filler Concentration, Aspect Ratio and Orientation on the Electrical Response of Carbon Nanotube/Polymer Nanocomposites. *Compos Sci Technol* **2011**, *71* (5), 643–646. https://doi.org/10.1016/J.COMPSCITECH.2011.01.005.

(5) Lloyd, E. C.; Dhakal, S.; Amini, S.; Alhasan, R.; Fratzl, P.; Tree, D. R.; Morozova, S.; Hickey, R. J. Porous Hierarchically Ordered Hydrogels Demonstrating Structurally Dependent Mechanical Properties. *Nature Communications 2025 16:1* **2025**, *16* (3792), 1–9. https://doi.org/10.1038/s41467-025-59171-w.

(6) He, S.; Liang, W.; Tang, Y.; Zhang, J.; Wang, R.; Quan, L.; Ouyang, Y.; Huang, R.; Dou, R.; Wu, D. Robust Super-Structured Porous Hydrogel Enables Bioadaptive Repair of Dynamic Soft Tissue. *Nature Communications 2025 16:1* **2025**, *16* (3198), 1–12. https://doi.org/10.1038/s41467-025-58062-4.

(7) Alegret, N.; Dominguez-Alfaro, A.; González-Domínguez, J. M.; Arnaiz, B.; Cossío, U.; Bosi, S.; Vázquez, E.; Ramos-Cabrer, P.; Mecerreyes, D.; Prato, M. Three-Dimensional Conductive Scaffolds as Neural Prostheses Based on Carbon Nanotubes and Polypyrrole. *ACS Appl Mater Interfaces* **2018**, *10* (50), 43904–43914. https://doi.org/10.1021/ACSAMI.8B16462/SUPPL_FILE/AM8B16462_SI_001.PDF.

(8) Lee, C. S.; Kim, S.; Fan, J.; Hwang, H. S.; Aghaloo, T.; Lee, M. Smoothened Agonist Sterosome Immobilized Hybrid Scaffold for Bone Regeneration. *Sci Adv* **2020**, *6* (17). https://doi.org/10.1126/SCIADV.AAZ7822/SUPPL_FILE/AAZ7822_SM.PDF.
